# Problem-Based Learning Case of Unvaccinated Child With Measles Infection: Integrating Viral Pathogenesis, Immunology, and Vaccine Ethics

**DOI:** 10.15766/mep_2374-8265.11577

**Published:** 2026-02-06

**Authors:** Emily C. Onello, Andrew Skildum, Janet L. Fitzakerley, Benjamin L. Clarke

**Affiliations:** 1 Associate Professor, Department of Family Medicine and Biobehavioral Health, University of Minnesota Medical School Duluth Campus; 2 Assistant Professor, Department of Biomedical Sciences, University of Minnesota Medical School Duluth Campus; 3 Associate Professor Emeritus, Department of Biomedical Sciences, University of Minnesota Medical School Duluth Campus; 4 Professor, Department of Biomedical Sciences, University of Minnesota Medical School Duluth Campus

**Keywords:** Problem-Based Learning, Measles, Virology, Immunology, Vaccine Hesitancy, Immunization, Bioethics, Global Health

## Abstract

**Introduction:**

The resurgence of vaccine-preventable viral diseases and the emergence of novel viruses demand that practicing physicians understand viral infections, human immunity, and the impact of immunization and vaccine hesitancy on disease outbreaks. Teaching medical students these concepts is a crucial element of foundational medical education.

**Methods:**

We used a problem-based learning (PBL) case involving a toddler who presents at a rural clinic following international travel to integrate basic and clinical science. During the assigned five curricular hours, first-year medical students were expected to analyze data and create a detailed timeline of viral infection in order to make the diagnosis of active measles, and to construct concept maps that described the pathophysiologic, immunologic, and ethical aspects of the case. Students received formative feedback on their concept maps, and content experts used multiple-choice questions (MCQs) and subsequent item analysis to assess student knowledge regarding the case educational objectives.

**Results:**

We delivered this PBL case to 687 medical students as part of our required first-year curriculum from 2014 to 2023. Students achieved faculty standards of performance (≥70% correct responses) in 10 of 16 MCQs analyzed in this report, and 15 of 16 questions had a positive discrimination index.

**Discussion:**

This PBL teaching case is timely and adaptable, with heightened relevance during the COVID-19/SARS-CoV-2 pandemic and recent measles outbreaks. It is effective for teaching medical students the basic science of viral infections and the ethical and public health implications of vaccine hesitancy.

## Educational Objectives

By the end of this activity, learners will be able to:

1. Contrast the characteristics of regional, national, and international measles outbreaks, including a comparison of the rates of long-term adverse events.
2. Explain the route of entry and sites for viral replication during the prodromal stage of measles infection as they relate to the mechanisms of innate immunity.
3. Differentiate among the pathophysiologic processes that cause each of the clinical symptoms and signs experienced by a typical measles patient as the infection evolves over time.
4. Characterize the following elements of adaptive immunity: (a) the relationship between major histocompatability complex (MHC) class I expression and cell-mediated toxicity; (b) the separate roles for dendritic cells, lymphocytes, natural killer T cells, and B cells in combating measles virus; (c) the role of MHC class II expression in long-term humoral immunity; and (d) the immunologic functions of antibodies and the timing of the emergence of IgM, IgA, and IgG molecules during infection.
5. Describe the impact of herd immunity on the estimation of infectivity (R value) and on the spread of measles in a population.
6. Explain how the ethical principles of beneficence, nonmaleficence, autonomy, and justice apply to vaccination decisions, and apply these four principles when communicating effectively with patients and their families about vaccines.
7. Outline the mechanism of action for established measles treatments, focusing on acetaminophen's antipyretic and analgesic actions, vitamin A's effects on mucosal immunity, and the timing for administration of immune globulin therapy.

## Introduction

Measles is a highly contagious disease whose symptoms can go unrecognized by providers who lack sufficient training.^1–3^ The US declared measles eradicated in 2000,^4,5^ due to widespread MMR immunization. However, as vaccine hesitancy grows, more of the US population is at risk of infection from endemic sources.^6^ For example, the 2024–2025 US measles outbreak spread quickly across multiple states, with tragic and preventable health consequences.^7^ As old viral diseases resurface and new ones emerge, physicians must understand viral pathogenesis, clinical presentation, and disease management. Measles serves as a timely and relevant model disease to introduce medical students to the basic science principles related to viral infection and immunity, as well as the ethical and public health concerns related to vaccine hesitancy.

As of April 2025, the only *MedEdPORTAL* publication describing a curriculum focused on measles management is one that describes a vaccine-hesitancy curriculum for residents.^8^ A similar search of PubMed for the term *measles problem-based learning* (PBL) identified four publications describing health care education curricula, none of which were suitable for use in undergraduate medical education.

This report describes a validated learning exercise centered on the case scenario of an unvaccinated child with measles. This PBL case was adapted from real events involving a rural US pediatric measles case and associated parental decisions regarding measles vaccinations, with details changed to protect patient confidentiality and to reinforce learning goals. PBL is a well-established method for teaching concept-mapping skills^9–11^ to develop disease-specific knowledge, and to more broadly enhance clinical reasoning and presentation skills.^12,13^ The case fills a critical need in medical education curricula using a validated active learning strategy.

This measles case was one component of a suite of immunology and vaccine-related course elements. The educational impact of this suite has been published previously,^14^ with results showing significant increases in student self-rated knowledge and skill in talking with patients about vaccinations and immunology. While this earlier publication did not report on the isolated impact of the PBL case, the overall favorable findings provide evidence regarding the educational value of this PBL case. The aim of this report is to present a well-vetted curricular element that other medical educators may adopt.

## Methods

### Background

This case scenario (Appendix A) describes a partially immunized toddler who presents to a rural family medicine clinic after international travel. The case follows the patient and an unvaccinated cousin, culminating with the patient returning for a prekindergarten physical, still missing several required immunizations. Clinical and basic science faculty content experts developed the educational objectives, collaborated to write and review multiple-choice questions (MCQs; Appendix B) evaluating student performance according to each educational objective, and offered formative feedback to the students regarding their final concept maps. Session facilitators were primarily basic scientists who had 6 to 32 hours of training and/or experience in the PBL approach, but not specific expertise in viral diseases or immunology.^15^

This case was designed for first-year medical students as part of an immunology and hematology course, which was a required course of the University of Minnesota Medical School Duluth Campus (UMMS-DC) undergraduate medical curriculum. Prior to delivery of this PBL case, students were exposed to several fundamental concepts related to immunology, virology, ethics, and patient presentation via lectures. At the point in the curriculum when this case was delivered, students had already developed skills in the PBL process by participating in 5–10 other cases.

All UMMS-DC PBL cases shared the common goals of improving student proficiency in: (1) developing clinical concept maps, (2) presenting clinical information, and (3) working in teams (Appendix A). Students received formative feedback on these common objectives from their facilitators during this and all other PBL cases in the curriculum. A quick overview guide for delivery of this PBL case is provided in Appendix C, with more detailed faculty instructions offered in Appendix D.

### Implementation

#### Materials

• Electronic educational delivery platform format (e.g., Canvas) or paper version of the case
• Meeting space for groups of 6–8 students
• Colored marker set for each small group
• Large-group classroom space for review session with white boards
• Two students with laptop, tablet, or other device for part 2 (student session A)
• Each student with personal laptop, tablet, or other device for part 3 (student session B)

#### Personnel

• Small-group facilitators, 1 per group
• Faculty content experts, to review each case educational objective, assess student competencies, and provide formative feedback with respect to specific educational objectives

#### Part 1, faculty case preview (1 hour)

Content experts and the course directors reviewed the case annually to update the educational objectives and case details. Facilitators attended a 1-hour preview session where they provided feedback on the proposed changes and received reminders about the aspects of the case to emphasize. Facilitators were expected to be familiar with previous instruction that students had received regarding immunology and vaccine hesitancy, including the CASE framework for discussion of vaccines.^16^ This preview session was focused on quality improvement, and ensured that the case was focused and ran smoothly. It typically did not significantly alter the content as designed by the content experts.

#### Part 2, student session A (2 hours)

With guidance from their facilitators, student groups worked through the case, identifying new clinically relevant information from each page. At each stage, groups developed and modified testable medical hypotheses and student educational objectives. Only two students were allowed computers/devices to serve as fact checker and recorder, respectively. Limiting computers promoted interaction and dialogue. By session's end, students had developed a draft concept map (which included an indication of gaps in knowledge) and a list of student-generated educational objectives that they researched and incorporated into their concept map before the start of the next session.

As part of this session, students were asked to role-play a conversation regarding vaccinations and vaccine hesitancy among parents and a physician.^16,17^ The role-play exercise was informal and improvisational; a specific script was unnecessary. Most first-year medical students had already encountered vaccine-hesitant people and were familiar with many of the common concerns.^14^ Following the exercise, the group discussed effective and ineffective approaches to counter the parents’ vaccine hesitancy and incorporated these approaches into their concept map.

As homework between session A and session B, students studied their learning issues using textbooks, review articles, and primary literature.

#### Part 3, student session B (2 hours)

Three activities occurred during this session. First, a student presented a short (3–5-minute) case summary, using SNAPPS (Summarize, Narrow, Analyze, Probe, Plan & Select).^18^ Second, the students presented their concept map to each other, with each student communicating the information that they researched and facilitators providing formative feedback on the quality of their map (Appendix D). Last, facilitators provided students with the faculty core educational objectives (Appendix A) and two topical review articles^19,20^ for students to study in preparation for the final session (part 4).

#### Part 4, concept map review (wrap-up) session (1 hour)

As no facilitator can be an expert in all educational objectives^15^ and wrap-up sessions have been shown to have benefit in teaching generic concept-mapping skills,^21^ one faculty-selected group presented their final concept map to the class, facilitators, and content experts. Differences among the student maps were discussed in this large-group setting, and students were encouraged to ask questions about any aspect of the case, as well as to discuss and debate ethical concepts. Content experts emphasized important points and corrected any significant errors in either the organization or educational objective–related details of the presented map, which served as a second formative assessment of the students’ mapping skills.

#### Student assessment

Facilitators provided formative feedback to the group members at all stages of the concept-mapping process, generally commenting on the appropriateness of student learning issues, and encouraging students to create a robust concept map with logical connections between pathologic processes, symptoms, and therapeutic interventions.^22^ Content experts used MCQs and subsequent item analysis to assess student competency with respect to the case educational objectives. Questions assessing PBL content appeared on midterm tests, as well as on comprehensive final exams. The maximum time from the PBL to MCQ assessment was 4 weeks.

### Data Analysis

The University of Minnesota Institutional Review Board (IRB) determined that this scholarly activity is not research involving human subjects (IRB no. MOD00037898; March 13, 2023). The IRB was not involved before the authors decided to publish the case. We reviewed student concept maps from the past 5 years to develop the qualitative list of student challenges described under the concept map success section. We used quantitative analysis of response data from graded course assessments to evaluate the effectiveness of this PBL case (Appendix B). We did not include a preassessment of student competency.

To analyze the MCQ responses, we examined the most recent versions of the 16 most frequently used test questions between 2015 and 2023. This corresponds to the time interval that ExamSoft was used as the testing platform. We used Excel to perform the statistical analyses of ExamSoft item analysis response data (complete data for each question are found in Appendix B).

## Results

Six hundred eighty-seven first-year medical students participated in this measles PBL exercise and took the summative assessments between 2014 and 2023, spanning 10 academic years.

### Formative Concept Map Assessment

**Figure 1.**
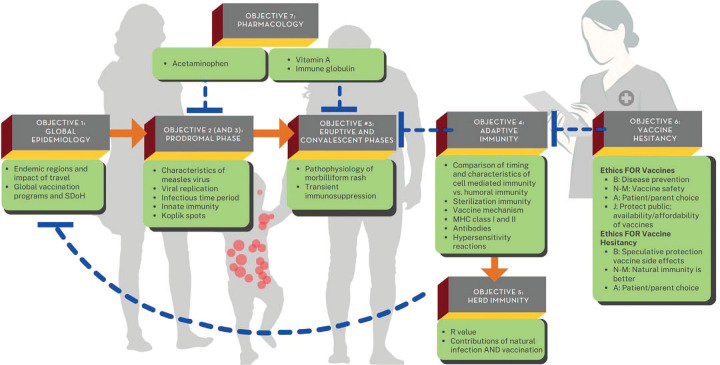
Content expert overview of measles concept map organization. Student scientific concept maps should be organized into seven major interlinked sections, each corresponding to a specific educational objective for the case. The distribution of key topics (indicated as bullet points) is not evenly divided among the objectives, as adaptive immunity (educational objective 4) is the core focus of the case and should be integrated across several sections of the map. Actual student scientific concept maps should contain much greater detail on each key topic. Orange arrows indicate causation; blue blunt-ended dashed lines indicate inhibition. Abbreviations: A, autonomy; B, beneficence; J, justice; MHC, major histocompatibility complex; N-M, nonmaleficence; R value, R-naught or R0 value for communicable diseases; SDoH, social determinants of health.

The concept maps were assessed during both the third (part 3, small group) and fourth (part 4, entire class) sessions by the facilitators and content experts, respectively, with the goal of improving student proficiency in developing clinical concept maps, the first of three UMMS-DC PBL learning goals (Appendices A and D). The expectation was that student concept maps would be divided into seven major interlinked sections corresponding to each specific case educational objective (Figure 1). The distribution of key topics (i.e., concept links, indicated as bullet points in Figure 1) was not evenly divided among the objectives, with the expectation that adaptive immunity (objective 4) would be the core focus of the case and should be integrated across several sections of the map.

By this stage of their medical school journey, students had significant concept-mapping skills. Most were capable of rearranging information from the clinical timeline (presenting concern, history, labs, etc.) to a viral pathophysiologic process (infection in the population → patient infection/prodromal signs → eruptive and convalescent phases, etc.).

**Figure 2.**
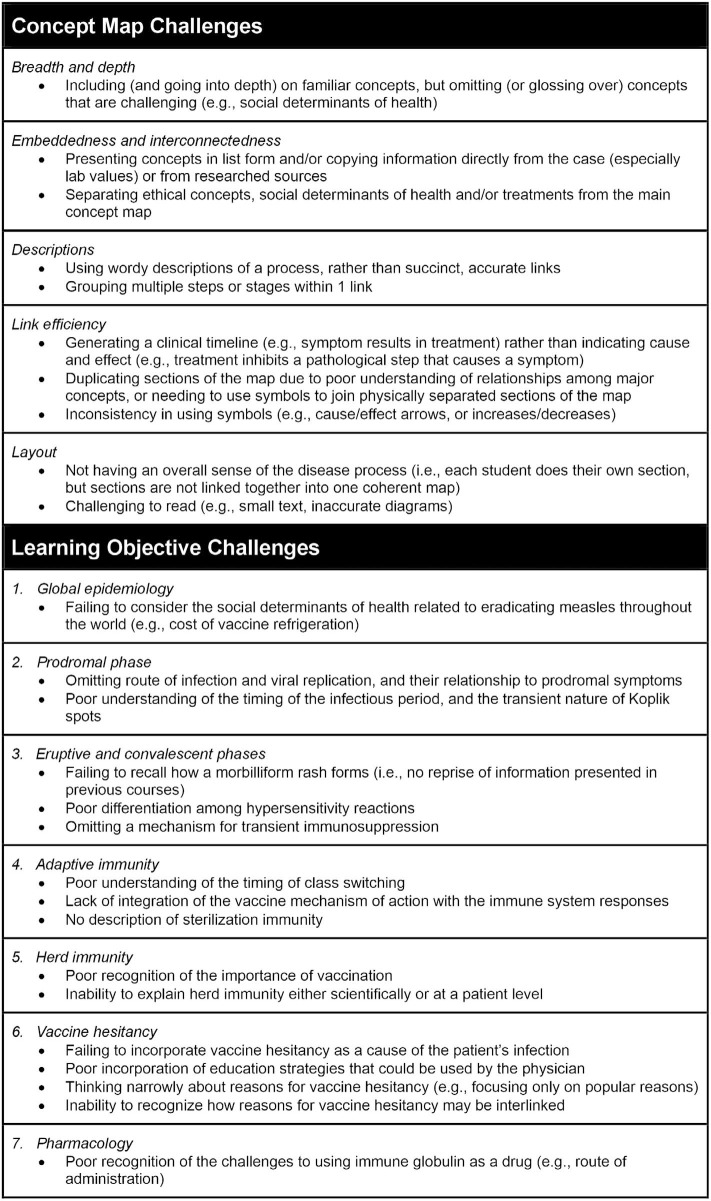
Common student challenges in concept mapping, summarizing common areas where medical students were challenged to meet faculty expectations for a thorough and integrated concept map and/or an adequate exploration of the measles problem-based learning case educational objectives. The authors used a 5-year retrospective review of student concept maps along with facilitator recollections to identify these common challenges.

There were several areas where students were challenged to meet faculty expectations (Figure 2), with significant variability among groups with respect to the topics that presented the most difficulty. Students found integrating treatment, herd immunity, and vaccine hesitancy the most consistently challenging. The degree of challenge changed across the years, as content experts provided more data. For example, findings from enzyme-linked immunosorbent assay (ELISA) tests (which are not typically seen by a physician) were added to prompt the students to include more details regarding IgG/IgM class switching in their concept maps. Similarly, the inclusion of the serum cytokine panel encouraged students to consider the roles of distinct types of lymphocytes.

### Summative Assessment

Student performance on a particular version of each MCQ was consistent from year to year, with improvements in student performance being observed when questions were revised and/or when the case content was updated to address the educational objective more specifically.

**Figure 3.**
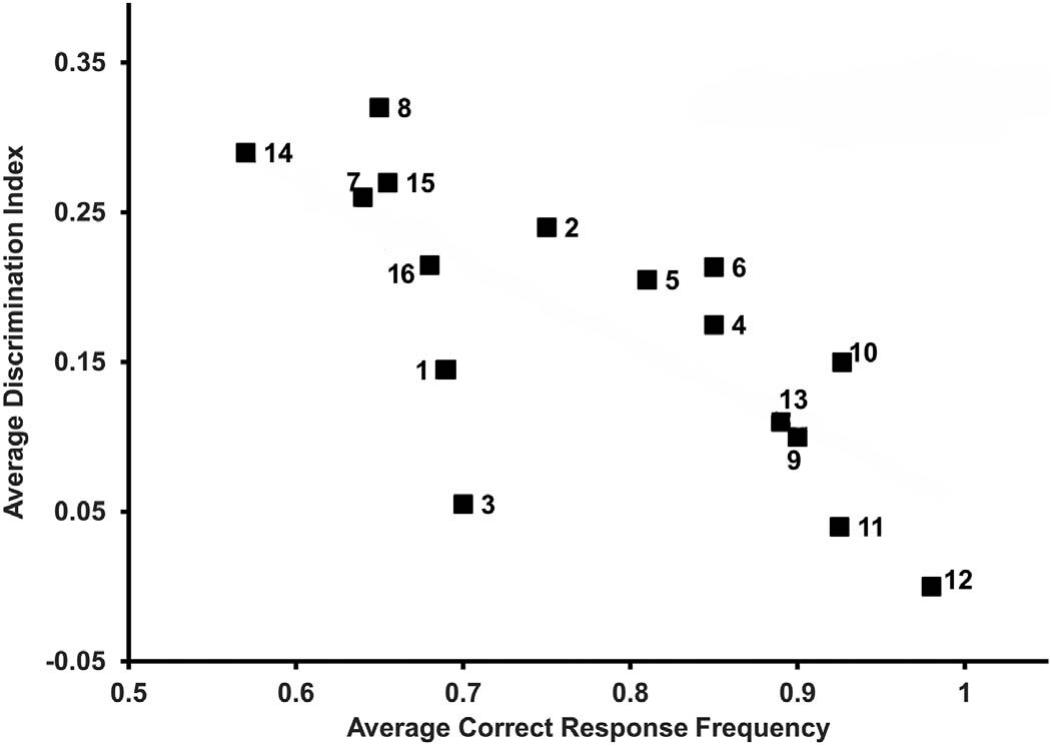
Question metrics for the 16 multiple-choice questions evaluating student performance on the measles problem-based learning case from 2014 to 2023. Mean correct-response frequencies (x-axis) were calculated as the fraction of total correct responses divided by the total number of responses (i.e., 70% correct = 0.7). Mean discrimination indices (y-axis) represent the difference in item performance between top- and bottom-performing students, with positive values indicating that students who did well on the overall test performed better on the question. Numbers adjacent to the data points indicate the question numbers, as provided in Appendix B. The appendix presents raw data, including sample sizes.

As shown in Figure 3, student correct-response rates on the 16 MCQs showed a mean student performance ranging from 52% to 98% after participation in the measles PBL case, with the best performance being on the question related to herd immunity (educational objective 5). The students collectively achieved the faculty standard of 70% (0.7 criterion) in 10 of 16 MCQs and scored above 90% (0.9 criterion) on 3 of 16 MCQs. A total of 15 of 16 MCQs had a positive mean discrimination index, indicating that top students (those who performed well on the overall test) performed better on those 15 questions. There was a negative relationship between the discrimination index and correct-response frequency, as has been observed in other studies.^23^ Mean discrimination index values were between 0 and 0.3 (raw data and sample sizes for each question are provided in Appendix B). The most-challenging questions for the lower-performing students were the questions regarding the generation of symptoms in the prodromal phase (educational objective 3; questions 7 and 8), vaccine hesitancy (educational objective 6; questions 13 and 14), and pharmacology (educational objective 7; questions 15 and 16), as indicated by the higher discrimination index values.

## Discussion

When this PBL was launched in 2014, there was skepticism among the course faculty about the utility of spending 5 hours of curriculum time on a case involving a virus considered eradicated from the US. However, this measles PBL activity has proven to be an ideal showcase for exploration of common principles of viral infection and the human immune response that are applicable to other viral infections. The case also provided a rich context for students to discuss the clinical implications and changing ethics of vaccine hesitancy^1,6^ during several outbreaks of vaccine-preventable diseases (including measles and COVID-19). In recent years, outbreaks of viral diseases occurred in the US around the time that this measles PBL case was delivered. Notably, in 2017, two outbreaks were featured in the local media: a measles outbreak within Minnesota, and a mumps virus outbreak involving the University of Minnesota Twin Cities campus. Student knowledge, as assessed by their MCQ responses, may have been influenced by the public health themes presented in the media and discussed broadly in the community. These public events favorably shifted the perception of the students and faculty regarding the utility of this case.

The strengths of this PBL case include the active learning format and the opportunity for students to work collaboratively while developing a concept map of the underlying disease process. These are features of the deliberate practice model of the development of professional expertise.^24^ The summative assessment data support the use of this PBL as an effective learning tool to address the educational objectives developed by the faculty. This case also challenges students to consider how they would professionally handle a time-sensitive reportable infectious disease outbreak in their own primary care clinic.

The limitations of this PBL case include the considerable time commitment, as well as the need for content experts, trained faculty facilitators, and adequate small meeting room space. In addition, there is unavoidable variability among groups as it is impossible for all small groups and facilitators to discuss and emphasize precisely the same concepts, or for students to have the same background knowledge. In addition, the concept map review session was contentious, partly because students disliked the pressure of presenting to a large group, and also because some facilitators felt that students were more passive during session B (part 3) when they knew that the correct information would be presented in the review session (part 4). These final review sessions were eliminated from the curriculum in 2020 when COVID-19 restrictions prevented large live sessions, and they were not reintroduced.

This report presents the experience of a single institution (UMMS-DC), which has a small class size (60–65 students). The generalizability for other medical schools remains untested, though the UMMS has recently expanded the use of PBL across campuses for a combined annual enrollment of 245 students. The personnel and space requirements to deliver this PBL case may present significant challenges for institutions with larger class sizes. However, this case could translate well into other delivery formats, such as a case study. It may also be necessary to modify the case if students or faculty members are unfamiliar with concept mapping. In such cases, instructors should consult published summaries of how to use concept mapping in medical education.^25^

We have shown that this PBL measles case curriculum is both valuable and adaptable in promoting the integration of ethics and social science with basic science competencies. It has been time-tested and provides an active learning opportunity for health professional training programs seeking a realistic and engaging clinical case.

## Appendices


Faculty Guide.docxExam Questions.docxHow-to-Deliver Quick Guide.pdfHow-to-Deliver Full Guide.pdf
*All appendices are peer reviewed as integral parts of the Original Publication.*
