# Supplementary material for: Problem-Based Learning Case of Unvaccinated Child With Measles Infection: Integrating Viral Pathogenesis, Immunology, and Vaccine Ethics
Source: MedEdPORTAL. 2026 Feb 6;22:11577. doi: 10.15766/mep_2374-8265.11577 (PMC12890053; doi:10.15766/mep_2374-8265.11577)
Supplement: Supplementary file 1 — Faculty Guide.docxExam Questions.docxHow-to-Deliver Quick Guide.pdfHow-to-Deliver Full Guide.pdf [file mep_2374-8265.11577-s001.zip › A. Faculty Guide.docx]

### Problem Based Learning (PBL) Case of Unvaccinated Child with Measles Infection: Integrating Viral Pathogenesis, Immunology and Vaccine Ethics

**APPENDIX A: FACULTY/FACILITATOR GUIDE**

*Contents*

General Instructions Page 2

Student session A Page 3

Student session B Page 23

Concept map review (“wrap up”) session Page 25

References Page 26

**GENERAL INSTRUCTIONS**

This document contains both the case content that is presented to the students (black ink) and the facilitator’s notes (blue ink). All of the blue ink facilitator notes should be removed to create the student version.

Note that aspects of this case are specific to Minnesota, USA and may need to be adjusted for the location of the medical school (for example, immunization requirements for school attendance, options for vaccine exemptions, treatment during active infection, quarantine requirements, etc.).

This case is designed for printing but could also be presented using an online learning platform. Whether using printed or digital version, the case should be presented to the students one section at a time, with students following the prompts to ensure that their concept map incorporates all of the objectives identified by the content experts.

Facilitators should attend a one hour preview session run by the course director(s) and case content experts. During this session, everyone reads through this guide together so that they can clarify any questions or concerns and make suggestions for improving the case or its delivery.

Students will need to work together and stay on task to complete the sessions on time. Typically, there is one group leader who reads the case, ensures that all of the prompts are followed and makes decisions for the group. One person (scribe) is responsible for organizing the data, the timeline, and the concept map on the whiteboard. One person (recorder) takes digital notes in a common storage space (e.g., a school monitored Google drive space), paying particular attention to the group’s learning objectives. The other members of the group must assist as needed to ensure that all of the information in these case notes is included in their final concept map.**STUDENT SESSION A**

The sections in this case are:

1. Presenting Concern (15 minutes)
2. History of the Present Illness (10 minutes)
3. Past Medical History (10 minutes)
4. Physical Exam Findings (10 minutes)
5. Laboratory Tests (10 minutes)
6. Clinical Course (10 minutes)
7. Clinical Course, continued (10 minutes)
8. 3 Month Follow up (10 minutes)
9. 2½ Year Follow up (30 minutes; no more than 20 minutes for role play)

Suggested time to be spent on each section is indicated in parentheses. Each group may progress at a slower or faster rate than indicated for each section, but everyone must complete all work by the end of the scheduled time. It is expected that documenting the data and modifying the group concept map will be the primary focus of each section.

Facilitators should ensure that they are familiar with the ethical issues surrounding vaccines^1^, the CASE approach to dealing with vaccine hesitancy^2^ and with the two review articles that students will be required to read before the concept map review (“wrap up”) session.^3,4^

This PBL case is rich with opportunity for students to explore the ethics of beneficence, non-maleficence, autonomy, and justice as they apply to vaccine decisions:

BENEFICENCE: Positive impacts of a healthcare intervention on individual patients, as well as on the collective well-being of the community. For example, administration of a vaccine decreases the severity of illness in individual patients, as well as contributing to herd immunity.

NON-MALEFICENCE: “First, do no harm”. For example, a vaccine should not be given to a patient who has a medical contraindication.

AUTONOMY: The right of competent people to make informed decisions about their own medical care. For example, parents have the right to decide whether their children are vaccinated.

JUSTICE: The fair and equitable distribution of resources and care, and/or ensuring that patients are not denied care due to bias or inequalities in the distribution of scarce resources. For example, rural patients may not have the same access to the vaccinations as patients in more urban locations.

This case includes specific, structured opportunities for discussion of these ethical principles, particularly during the past medical history, as well as the 3 month and 2 ½ year follow up visit sections. However, it is common for students to raise ethical issues at other points in the case, for example, during the history of the present illness or during discussion of the contact tracing information presented during the clinical course sections.

In these ethical discussions, two common challenges for students are:

- Focusing their discussion on the physicians’ ethical perspective, and not on the parents’. For example, students can spend time arguing that parents who choose not to vaccine their children are violating the principle of non-maleficence, as they are imposing risks of harm on non-consenting 3^rd^ parties. Instead of dwelling on this discussion, facilitators should encourage the students to focus on the principle of autonomy of vaccine choice from the physician’s perspective.
- Determining which “patient” a physician should protect. Physicians have a responsibility to the other patients in their practice, as well as to the individual patient and to the general public. Understanding how the ethical principles interact in decisions involving these different responsibilities --- and that there are no absolute rules --- are keys to achieving learning objective 6.

**PRESENTING CONCERN:**  19 month old patient, Brian, presents for new onset fever and rash

**Document** the new data you have, including a detailed timeline of any symptoms.

To identify gaps in your knowledge, **formulate questions** based on these new data. Questions must be open ended – i.e., they cannot have a yes or no (closed) answer. Samples of open questions are as follows:

(WI) What is_________________________?

(WIS) What is the significance of ______________________?

(WIRB) What is the relationship between _________ and __________?

(WIM) What is the mechanism for _________________?

(WAC) What are the causes of __________________?

**Develop hypotheses**. Your hypotheses should link a disruption in a normal physiological process to at least one symptom experienced by the patient. Consider all possible systems, including social determinants of health, when developing your hypotheses.

Document **learning issues** that you will research before the next session. Be prepared to discuss these learning issues at the next group session.

**Discuss** questions you would ask the mother. How would information that you gain from taking a careful history be used to evaluate the hypotheses under consideration?

**FACILITATORS’ NOTES**

Critical information on the first page:

- Objective 2: Prodromal phase
- Objective 3: Eruptive and convalescent phases

Prompt students to recall pathophysiological mechanisms for fever and rash(es). Make sure the students begin work on a **detailed timeline**for the infection, including approximate viral exposure (while traveling abroad), onset and sequence of symptoms and contagiousness, all the way through convalescence and long term immunity.

**HISTORY OF PRESENT ILLNESS**

Brian and his family recently traveled to India and Malaysia on vacation; they returned to the United States one week prior to this presentation at the clinic. While traveling overseas, they report drinking only bottled water. However, they recall that there was an outbreak of diarrhea on an island off the coast of Malaysia where they were staying. They returned to the United States through the Los Angeles airport; from there they traveled to Lincoln, Nebraska to visit relatives for two days, then returned to Minnesota.

The day after they returned to their home in Grand Timber, MN, Brian seemed particularly clingy and low-grade fevers were noted. He continued to breastfeed around the clock but avoided solid foods. Brian’s appetite continues to be diminished. He continued to make urine and tears. No mental status changes were noted, aside from increased irritability. No rigors or lethargy were observed.

Over the last 48 hours, axillary temperatures at home were as high as 103°F, worse in the evenings. He developed some clear rhinorrhea, watery eyes, and a dry cough; wheezing or dyspnea were not observed. Within the last 48 hours, he had 2 episodes of non-bloody emesis after attempting solid foods (toast). His parents noted two episodes of diarrhea over this period as well. No mouth sores were observed. His parents have given intermittent acetaminophen (Tylenol).

Brian’s parents noticed a rash on his cheeks yesterday. When they saw the rash spreading to his body today, they became more concerned and chose to bring Brian to see the doctor. Before seeing the doctor today, they waited in the clinic waiting room. Several other patients, ranging in age from a 3 month old infant present for a well child visit to an 83 year old undergoing cancer treatment, were also seated in the waiting room at the time.

**Document** the new data you have.

To identify gaps in your knowledge, **formulate questions** based on these new data.

**Expand hypotheses**. Your hypotheses should link information from the history to a disruption in a normal physiological process that leads to at least one symptom experienced by the patient.

Document **learning issues** that you will research before the next session. Be prepared to discuss these learning issues at the next group session.

**Discuss** questions you have about the patient’s past medical history. How would information that you gain from taking a careful history be used to evaluate the hypotheses under consideration?

**FACILITATORS’ NOTES**

Critical information on this page:

- Objective 1: Global epidemiology
- Objective 2: Prodromal phase
- Objective 3: Eruptive and convalescent phases
- Objective 5: Herd immunity
- Objective 7: Pharmacology

Prompt students to develop pathophysiological mechanisms for **all** of the symptoms described on this page. Make sure the students update their **detailed timeline**for the infection, as this will help them resolve issues of cause and effect (effects cannot occur before causes).

If students develop hypotheses related to infectious diseases, suggest discussion of communicability, and more specifically what is the ‘R naught’ value for specific pathogens, or the ‘basic reproduction number’ for infectious diseases? Note that the students should not have a definitive diagnosis of measles at this point.

That said, understanding when patients are infectious and the high transmissibility of measles are critical components of this case. Students should take note of the fact that Brian was in close proximity to several other patients in the waiting room. At this stage, the implications of exposure to a generic respiratory pathogen should be discussed both from an infectious disease (R0) and from a public health perspective.

**PAST MEDICAL HISTORY**

Brian was born by spontaneous vaginal delivery at home with the aid of a midwife at 39-weeks-gestation following an unremarkable pregnancy. He was breastfed until he was weaned recently. His parents agreed to the state health department Newborn Screen which was completed when 5-days old; results were normal. He has had no significant illnesses, surgeries, accidents, or hospitalizations.

There have been no developmental concerns at well child visits thus far and Brian’s growth has been within normal limits (growth chart attached).

*One month Well Child visit:* Brian was healthy. Vaccinations were discussed and declined by parents. Parental rationale was to let his immune system develop naturally on its own.

*Two Month Well Child visit:* Brian was healthy. Vaccinations were discussed but declined by parents; they cited concerns about links between vaccination and autism.

*9 Month Well Child visit:* Brian was healthy. Vaccinations were discussed but declined by the parents.

*18-month Well Child visit:* Brian was healthy. Vaccinations were discussed in context of upcoming foreign travel plans. His parents agreed to IPV and DTaP only. Brian was administered DTaP and IPV at this visit, with boosters for both three months later.

**SOCIAL HISTORY:** Brian’s parents are married and educated through the college level. Both are non-smokers with no alcohol or illicit drug use. They have lived in rural Minnesota near Grand Timber for eight years. His parents are restoring an old historic building in their rural community. The family is omnivorous.

The family has health insurance from the father’s employer.

**FAMILY HISTORY:** No known hereditary illnesses are reported. Brian has no other siblings.

**MEDICATIONS:** Brian takes no regular medications. He has recently used over-the-counter acetaminophen.

**ALLERGIES:** Brian has no known allergies to medications, and no history of seasonal allergies is reported.

**Document** the new data you have. Construct a **timeline** that documents Brian’s past medical history, social history, and the onset of his current symptoms.

To identify gaps in your knowledge, **formulate open-ended questions** based on these new data.

**Expand hypotheses**. Your hypotheses should link information from the past medical and social history to potential disruptions in a normal physiological process that leads to at least one symptom experienced by the patient.

**Document learning issues** that you will research before the next session. Be prepared to discuss these learning issues at the next group session.

**Discuss** how you would approach Brian’s physical examination. How could information that you gained from a review of systems and physical examination be used to evaluate the hypotheses under consideration?

**List** the ethical considerations of beneficence and non-maleficence which apply to the administration of vaccines to children and **link** any relevant information to your draft concept map.

**Discuss** your responsibility (if any) to the other patients that were in the waiting room.

**
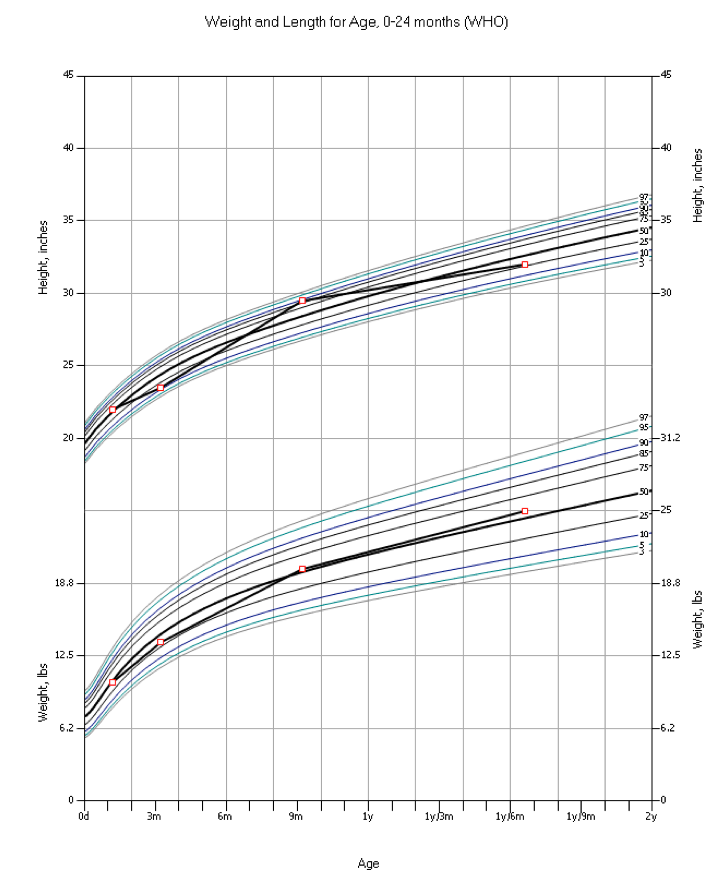
Figure A1:** Growth chart showing weight and length for age, 0-24 months.
Image by World Health Organization (WHO), retrieved in 2014. Image modified by author E.O using a free online child growth curve calculator. Image was in the public domain but is not accessible as of May 2025.

**FACILITATORS’ NOTES**

Critical information on this page:

- Objective 4: Adaptive immunity
- Objective 6: Vaccine hesitancy

Students should consider what happened during the vaccination and booster at the 18-month well child visit: Antigen presentation, B cell response, development of T memory cells, etc.

Students should recognize that the patient’s immunization status is incomplete and compare it to the current recommended immunization schedules. In particular, Brian has not received his first dose of the MMR vaccine

The recommended immunization schedules in the United States are updated annually. Students should identify and review the current recommended immunization guidelines as they discuss this case. The 2025 Immunization Schedule is provided below as an example for facilitators who may not be familiar with the immunization schedules.^17^

If students choose something like “Research the recommended vaccine schedule” prompt them to focus on the *rationale* for the schedule rather than memorizing details of what vaccines are given and when. For example, the MMR vaccine is first given at 12-15 months of age because maternal antibody levels have decreased to a point where the baby can mount a long-lasting immune response.

It is also important to identify the reasoning for refusal of vaccines, and to link these reasons to their mechanism, as this will help with later ethical discussions regarding beneficence, non-maleficence, autonomy, and justice. There are several places in the case where it would be appropriate for students to discuss vaccine hesitancy and to expand their mechanism to include ethical principles.

This can be a place where students discuss the ethics of denying care for patients who refuse vaccines, particularly if they recall that other patients were exposed in the waiting room. If they raise the issue, it is appropriate to ask students to discuss (and/or research) alternatives to banning patients from a practice, including the challenges to a “banning strategy” in rural communities where there are fewer options for care. This falls under the question of justice in the delivery of care.

**
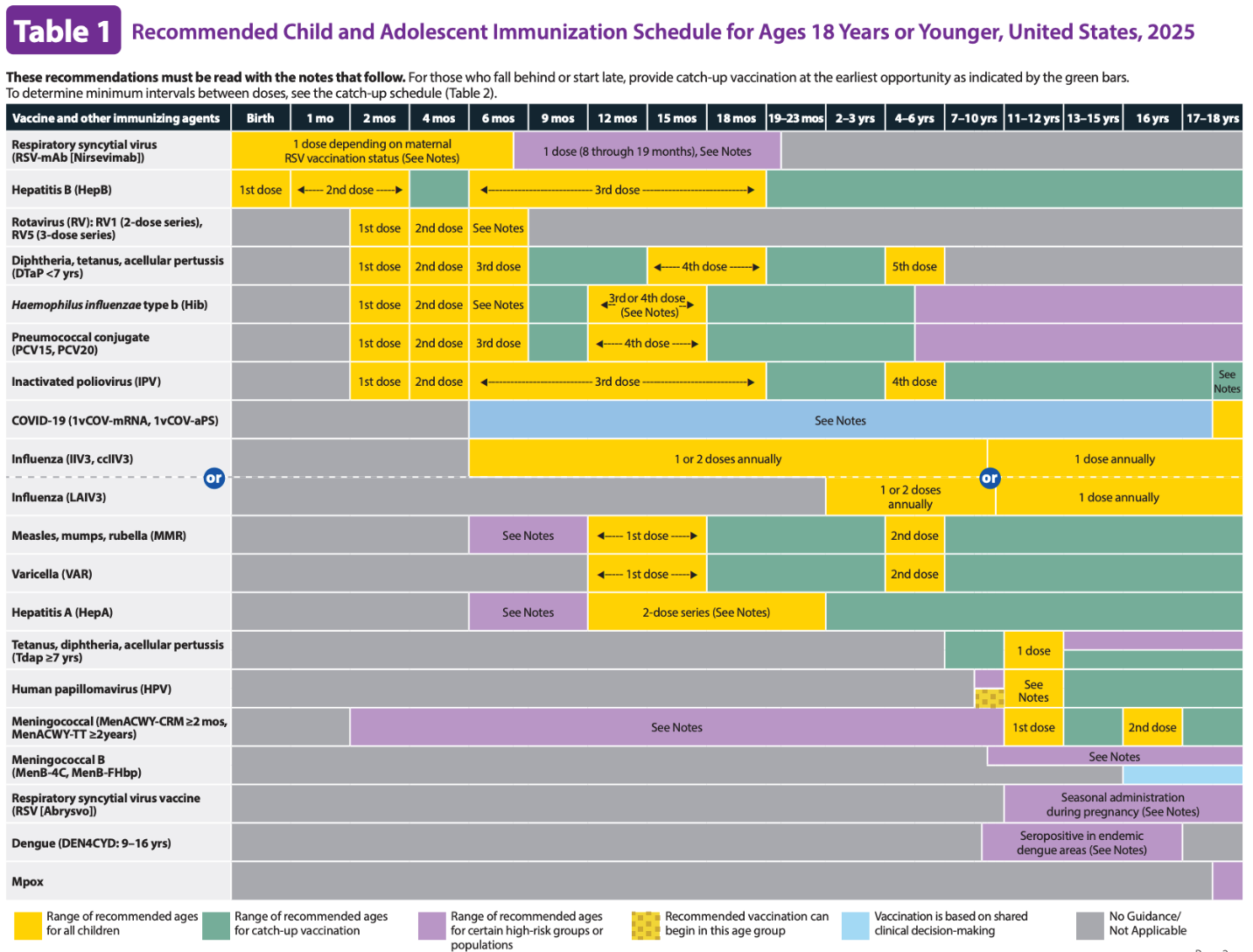
Figure A2:** Recommended child and adolescent immunization schedule for ages 18 years or younger, United States. This table is only intended as a reference for the facilitators (i.e., it should be removed from the student version of the case).

Image by U.S. Centers for Disease Control and Prevention (CDC), retrieved from <https://www.cdc.gov/vaccines/hcp/imz-schedules/downloads/child/0-18yrs-child-combined-schedule.pdf>, on July 26, 2025. Image is in the public domain.

**PHYSICAL EXAM FINDINGS**

***General appearance***: Brian appears alert, responsive, and irritable. He cries frequently during the exam when approached by the physician, but he is consolable in his parent’s arms. Tears are noted with crying.

***Vital signs***: Height: 34“, Weight: 25 lbs.

**Patient Value** **Reference Range**

Blood pressure 90 / 56 mmHg (89-104) / (44-58)

Heart rate 134 beats/min 80-150 beats/min

Respiratory rate 28 breaths/min 24-40 breaths/min

Temperature 102.9^o^F (40°C), rectal 97.4 – 99.6 ^o^F

***HEENT***: His neck is supple, tympanic membranes clear, no pain with movement of external ears. Extraocular muscles are normal; his eyes show a mild amount of clear discharge and some mild conjunctivitis. Brian’s oropharynx is unremarkable and without lesion; his mucous membranes are moist. No Koplik spots were observed. No nasal discharge is noted during the exam, nor any cervical adenopathy. Erythematous rash was noted; the rash was continuous around cheeks to his anterior and posterior neck. See skin exam described in section below.

***Lungs:*** Clear to auscultation without wheezes, crackles, or consolidation

***Cardiovascular***: Regular rhythm with no obvious murmur (difficult to assess due to short interval between heartbeats), peripheral pulses palpable

***Abdominal Exam:***  Appearance is normal, non-distended, normal bowel sounds heard, soft, non-tender, no hepato-splenomegaly.

***Genital Exam:*** Normal uncircumcised penis, descended testes without asymmetry or palpable abnormalities

***Neurological examination*:**  Grossly non-focal, cranial nerves intact, no meningeal signs

***Skin:*** Brian’s skin is supple; forearm skin does not tent when gently pinched by the examiner. Both cheeks are mildly erythematous. On front and back torso, a maculopapular, patchy, variegated, erythematous rash is noted along with additional discreet 1 mm papules extending inferiorly as far as the inguinal folds. Rash has morbilliform appearance (*see accompanying image*). No vesicles, petechiae, ecchymoses or purpura

***Extremities:*** Normal appearance without joint swelling or erythema, normal movement of limbs is noted


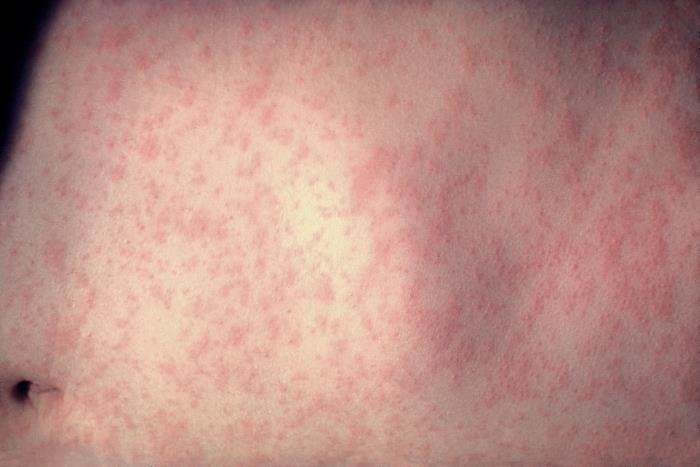


**Figure A3**: Skin rash on a patient’s abdomen 3 days after the onset of a measles infection.

Image by Eichenwald, H., retrieved from <https://phil.cdc.gov/Details.aspx?pid=3168> on September 23, 2025. Image was in the public domain.

**Document** the new data you have.

To identify gaps in your knowledge, **formulate questions** based on these new data.

**Expand hypotheses**. Your hypotheses should link new information from the physical examination to potential disruptions in a normal physiological process that leads to at least one symptom experienced by the patient.

Document **learning issues** that you will research before the next session. Be prepared to discuss these learning issues at the next group session.

**Discuss** what laboratory tests and procedures you would like to order. How could information that you gained from the labs be used to evaluate the hypotheses under consideration?

**FACILITATORS’ NOTES**

Critical information on this page:

- Objective 3: Eruptive and convalescent phases

Morbilliform rash is a descriptive term used that describes a “measles-like” rash. Pertinent findings include the rapid heart rate, elevated temperature, fussiness (while still consolable), absence of findings for dehydration or lethargy, absence of Koplik spots (see explanation below) , clear lungs, absence of heart murmur or other localized site of infection (no ear, lung, tonsillar infection). Key here is the development, classic for measles, of a diffuse erythematous rash that started on the face and cheeks and moved down the chest and back.

This rash is a type 2 and 3 hypersensitivity reaction; facilitators should prompt students to investigate the rash’s mechanism, if necessary.

Absence of tears with crying would suggest that a child is dehydrated. Therefore, the presence of tears is of clinical importance in the assessment for dehydration. Brian had diarrhea, which can cause dehydration.

Facilitators should ensure that students construct a detailed timeline that separates:

- the incubation phase (~7-14 days before symptoms appear)
- the appearance of prodromal symptoms (fever, runny nose, cough, Koplik spots) 2-3 days after symptoms begin
  - Note that Koplik spots appear transiently, and in only 50-70% of patients. It is, therefore, reasonable that Brian does not exhibit Koplik spots during the physical exam due to the timeline of this case (this is what occurred in the real life situation that this case is based on). Students may not think about the presence or absence of Koplik spots until they research the symptoms of measles after session A. Facilitators should be prepared to have a discussion of the influence of pathognomonic symptoms on a differential diagnosis at some point during these sessions (i.e., presence of Koplik spots is highly suggestive of measles, but absence of Koplik spots does not conclusively rule out measles). Facilitators should also ensure that Koplik spots are part of the group’s final concept map, with a scientific explanation of why they occur when they do.
- the rash (3-5 days after symptoms begin).

**LABORATORY TESTS**

Complete blood count: **Brian’s values Normal Ranges**

RBC 4.13 3.70 - 5.30 (x 10^6^/μL) Hematocrit 33.0 33 - 49 %

Hemoglobin 10.9 10.5 – 13.5 g/dL

MCV 80 70 – 86 (fL)

MCH 26.4 23 – 31 pg

MCHC 33.0 30 – 36 %

RDW 15.0 11.5 – 16.0 %

WBC 5.4 6.0 – 17.0 x 10^3^/μL

PMNs 44% 15 – 35 %

Lymphocytes 50% 45 – 76 %

Mono/Eos/Baso 6% 0 -12%

PLT 295 K 150 – 450 K/μL

MPV 10.0 6.5 – 10.0 fL

**Basic Metabolic Profile:** Within normal limits for electrolytes and kidney function

**Stool Bacterial Culture:**  Pending

**Stool Ova & Parasites:** No ova or parasites seen. No white blood cells seen. Specific tests ordered for cryptosporidium, Cyclospora and microsporidia; results pending.

**Rotavirus testing:** Negative

**Serum Cytokine Panel:**

IL-2: 350 pg/mL (98-190 normal range)

IL-4: 170 pg/mL (110-150 normal range)

**Throat Swab PCR for measles virus:** Positive for measles virus

**Nasopharyngeal Wash:**  Negative for influenza and RSV viruses

**Blood cultures:** Pending.


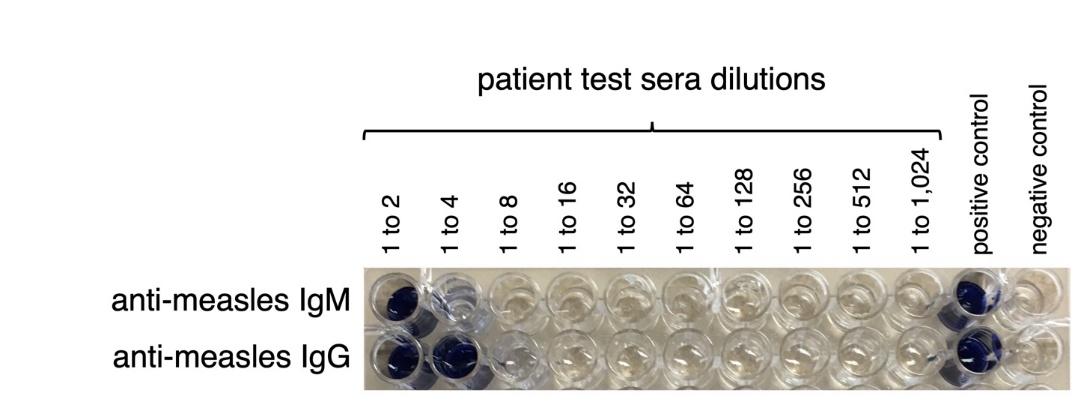
**Indirect ELISA Test for Measles Immunoglobulin:** Positive staining in wells less dilute than 1:16 are considered negative / inconclusive.

**Figure A4:** Indirect ELISA. Image is author owned.

**Document** the new data you have.

To identify gaps in your knowledge, **formulate questions** based on these new data.

**Expand hypotheses**. Your hypotheses should link abnormal laboratory values as either causes or consequences of disruptions in normal physiological processes that lead to at least one symptom experienced by the patient.

Document **learning issues** that you will research before the next session. Be prepared to discuss these learning issues at the next group session.

**Discuss** your recommendations for Brian’s treatment. What are the legal and ethical obligations of Brian’s physician to protect public health?

**FACILITATORS’ NOTES**

Critical information on this page:

- Objective 4: Adaptive immunity

Slightly diminished total WBC count: Measles is immunosuppressive (students may overlook this or find it surprising). The concept map should explain the immunosuppression.

Results of the initial ELISA test shown are negative for both IgM and IgG.

IgM antibodies typically appear 3-10 days following viral infection. The titer continues to rise for 2-3 weeks and then declines to undetectable levels after about 3 months. The presence of IgM specific antibody may indicate a current or recent acute infection. However, IgM responses during acute infection may be weak or absent. Students may discuss false positive rates of IgM as well.

If current infection is suspected, the laboratory suggests that a second IgG serum specimen be submitted in 2-4 weeks. It is recommended that a specimen be submitted to culture for measles virus or a second serum be submitted for IgG determination in 3-4 weeks.

**CLINICAL COURSE**

Given that Brian’s parents understand the potential complications of measles infection, and live close to the hospital, Brian is sent home for quarantine. His parents are instructed to return immediately if they observe any lethargy, decreased urine, poor eating, respiratory distress, or cough because Brian is at high risk of developing a secondary infection. Procedures to isolate Brian from other patients are provided in case he does need to return to the clinic. His parents are instructed to inform triage of his condition immediately upon entering the clinic or emergency department.

Brian is prescribed 200,000 IU vitamin A orally once per day for 2 days. Once Brian has recovered from this acute infection, it is recommended that he receive the MMR vaccine.

Public health authorities are notified of the diagnosis of measles virus, and they assist in contacting people who were exposed to measles.

Blood and stool bacterial cultures returned negative; stool parasite testing was also negative.

**Document** the new data you have.

To identify gaps in your knowledge, **formulate questions** based on these new data.

**Expand hypotheses**. Your hypotheses should link Brian’s treatment plan to the amelioration of Brian’s symptoms and to the lessening of his impact on public health.

Document **learning issues** that you will research before the next session. Be prepared to discuss these learning issues at the next group session.

**Discuss** your strategies for communicating with Brian’s parents and their community about vaccinations.

**Identify** the populations of people that will be contacted by the public health authorities and add this information to your concept map.

**FACILITATORS’ NOTES**

Critical information on this page:

- Objective 1: Global epidemiology
- Objective 2: Prodromal phase
- Objective 3: Eruptive and convalescent phases
- Objective 4: Adaptive immunity
- Objective 5: Herd immunity
- Objective 7: Pharmacology

Students should enhance the group’s timeline, making sure to indicate when Brian was contagious and recognize that others in the community have been exposed to measles. Students should recognize the risk to unvaccinated patients who were in the waiting room with Brian.

In this clinical scenario, the physician must contact the Public Health authorities, as measles infection is a reportable disease. Subsequently, all of the patient’s contacts during the infectious period will be notified (as well as contacts of the contacts!), including people on the passenger airline manifests on each of Brian’s flights from India and Malaysia, and anyone in the waiting room within two hours after Brian left. Per public health protocol, all people born prior to 1957 are assumed immune by natural infection and no further action is needed. Unvaccinated people born in 1957 or later who have been exposed to an infected person should receive measles immunization or immunoglobulin.

Students may choose to discuss the issue of justice with respect to the costs of contact tracing and post-exposure prophylaxis (PEP). In Brian’s case, thousands of people were exposed, and the resources available to trace and offer MMR or immunoglobin could represent a strain on public health resources. Students may struggle to appreciate the costs of these public health measures.

Students should consider why the patient still needs an MMR immunization despite having a measles virus infection i.e., to gain protection against the other viruses in the vaccine (mumps and rubella).

**CLINICAL COURSE, continued**

The next day in the clinic, Brian’s family physician receives a phone call from an Emergency Department Physician in Nebraska. A parent and child have arrived in the ED there, after the parent was notified by the Minnesota Department of Health to seek immediate medical attention. The health department told the parents that their unvaccinated 2-year-old daughter needs treatment after playing with our patient 5 days ago. The Nebraska physician is considering prophylactic vaccination and/or administration of immunoglobulin.

**Document** the new data you have. Add the cousin’s exposure and expected immune response to your ***detailed timeline.***

To identify gaps in your knowledge, **formulate questions** based on these new data.

**Expand hypotheses**. Your hypotheses should link the physical properties of the infectious particle with Brian’s travel history and his cousin’s symptoms.

Document **learning issues** that you will research before the next session. Be prepared to discuss these learning issues at the next group session.

**Discuss** your recommendations to the Nebraska physician for the cousin’s treatment.

**FACILITATORS’ NOTES**

Critical information on this page:

- Objective 2: Prodromal phase
- Objective 4: Adaptive immunity
- Objective 7: Pharmacology

Students should integrate a timeline for the cousin into Brian’s timeline of illness.

Make sure the students fully discuss the out of town "cousin" exposure, as this forces the students to focus on the concepts of passive immunity versus primary immunization. The exposed unvaccinated cousin is highly susceptible to measles but has missed the 72 hour post-exposure window where MMR immunization could be protective.

Instead, induction of passive immunity with IgG injection is recommended. Students should dive deeply into the nuanced discussion that patients treated with immune globulin (Ig), or blood products, should NOT receive MMR (measles-mumps-rubella) vaccine until 3-12 months later after Ig as the treatment may interfere with the desired immune response to the vaccine.

If learners need help developing recommendations for the cousin, facilitators should guide a discussion based on three key questions from the Ask Me 3 framework from the Institute for Healthcare Improvement^5^. These questions have been modified here to represent a provider’s perspective:

1. What is the main problem faced by the cousin and the cousin’s community?
2. What does the physician need to do to address the problem?
3. Why is it important for the physician to do this?

*Students may raise ethical concerns about patient confidentiality at this point.* Breaking patient confidentiality shows disrespect for a person’s autonomy and may cause harm both by causing stigma (the patient/parents being responsible for causing someone else’s illness) and breaking trust. This can be an important opportunity for students to discuss physician responsibilities, and how to balance individual patient needs with public health requirements and goals. In these ethical discussions, facilitators should remember that they are not to serve as content experts, but to guide students toward framing the discussion in concrete terms and to defining and researching their own learning objectives.

**3 MONTH FOLLOW UP**

Three months after his initial presentation, Brian returns for a follow up visit. He appears healthy, with no evidence of rash or any other measles symptoms. His parents do not report any concerns and state that he has a healthy appetite and lots of energy.

**Indirect ELISA Test for Measles Immunoglobulin:**

*Positive staining in wells less dilute than 1:16 are considered negative / inconclusive.*

**
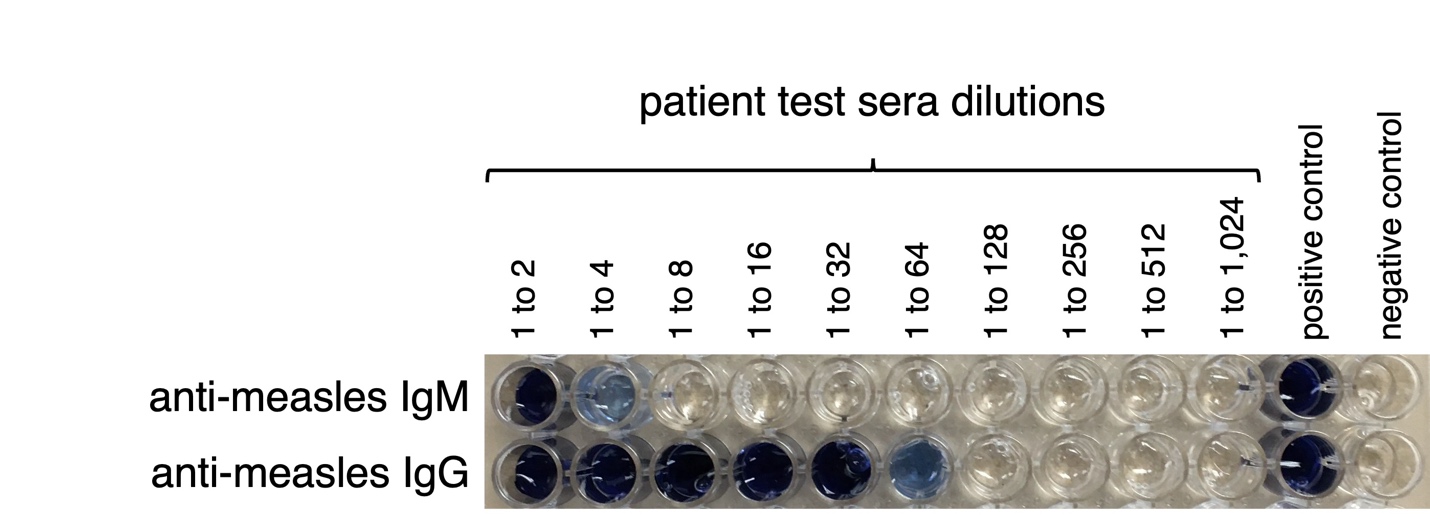
**

**Figure A5:** Indirect ELISA. Image is author owned.

**Serum Cytokine Panel:**

IL-2: 100 pg/mL (98-190 normal range)

IL-4: 300 pg/mL (110-150 normal range)

**Document** the new data you have. Add Brian’s cytokine and immune response to your ***detailed timeline.***

To identify gaps in your knowledge, **formulate questions** based on these new data.

**Expand hypotheses**. Add the processes of antibody generation and class switching to your mechanism.

Document **learning issues** that you will research before the next session. Be prepared to discuss these learning issues at the next group session.

**FACILITATORS’ NOTES**

Critical information on this page:

- Objective 4: Adaptive immunity

Results of this ELISA test 3 months later (convalescent serologic testing) are negative for IgM but positive for IgG.

IgG antibodies typically appear later than IgM antibodies and can remain detectable for longer periods of time. The presence of IgG when previously absent can be used to confirm a recent infection. The detection of anti-measles IgG can also be used to demonstrate immunity to measles.^23^

**2-½ YEAR FOLLOW UP**

You are seeing Brian for his pre-kindergarten Well Child Visit. Brian appears healthy and is developing normally, and his parents report no concerns. His primary vaccination series is not up to date, and no further vaccines have been given since the measles infection. Brian’s parents are adamant that they don’t want any vaccines. They ask his physician to complete the necessary vaccination paperwork required by the school.

**Document** the new data you have.

To identify gaps in your knowledge, **formulate questions** based on these new data.

**Expand hypotheses**. Add potential consequences of vaccine non-compliance to the health of Brian and his community to your concept map.

Document **learning issues** that you will research before the next session. Be prepared to discuss these learning issues at the next group session.

**Discuss** the physician’s legal and ethical obligation to certify that Brian is sufficiently immunized to attend school. Consider state law regarding exemptions for vaccination. For example, are the school requirements the same in Minnesota and Nebraska?

**ROLE PLAY** a conversation between Brian's parents and the physician. This role-play exercise can be informal and improvisational, and a rigid ‘script’ is not necessary. One student should take on the role of the mother, one student the role of the father, and one student the role of the physician. The remaining small group members are active observers. The ‘physician’ and ‘parents’ should express their concerns and goals.  What approaches are most effective for the physician, while respecting patient/parental autonomy? Add both these reasons for vaccine hesitancy and the physician approaches to your concept map. **THIS ACTIVITY SHOULD TAKE NO MORE THAN 20 MINUTES.**

**FACILITATORS’NOTES**

Critical information on this page:

- Objective 4: Adaptive immunity
- Objective 5: Herd immunity
- Objective 6: Vaccine hesitancy

During the role play, facilitators should encourage discussion about the incomplete vaccination status and whether or not Brian should attend nursery school. The students should consider the types of vaccine exemptions that exist (such as, medical, religious, philosophical) and determine which types apply in their location (they should realize that other places may have different regulations).

In addition, students should include a framing of the physician’s arguments from an ethical perspective (i.e., which of the 4 guiding principles is respected in each argument). For example, administering the vaccine to Brian would fall under beneficence; providing information about vaccines falls under informed consent (autonomy), etc.

Many first year medical students have already encountered vaccine hesitant people in their personal and/or professional lives^6^ and are familiar with some of the concerns about immunizations. As a result, students typically engage with this role playing exercise easily.

Facilitators should be prepared to remind students that they have learned the C.A.S.E. approach for talking to vaccine-hesitant patients and families^2^, and that the students playing the physician role should:

- Corroborate—Acknowledge the vaccine hesitant patient’s concern(s) and verbalize an understanding of why they might feel this way.
- About me—Move from acknowledging the concern to describing how they became educated on the issue.
- Science—Transfer key information that addresses the vaccine hesitant patient’s specific concern.
- Explain/advise—Discuss why they believe that immunization is important and in the best interest of the patient.

SAMPLE FACILITATOR PROMPTS FOR PROVIDERS:

- Do you fully understand the parents’ hesitation? What are their concerns, and where are they obtaining their information?
- Do you know why Brian has received some vaccines but not others?
- Do the parents understand the complications of measles?
- Do the parents know how vaccines work?
- Do parents understand the school exemptions?

SAMPLE FACILITATOR PROMPTS FOR PARENTS:

- What are you thinking about herd immunity and whether having a disease would impact the need for immunization?
- Do you think that “natural immunity” is better than vaccine-acquired immunity?
- What type of exemption are you seeking? Medical? Religious?
- Are you concerned about side effects? Short term pain? Flu-like symptoms? Autism?
- Do you believe that vaccine delivery should be staggered (i.e., only give one vaccine at a time)?

At the end of the discussion, students should determine whether or not Brian would be allowed to attend school in their location (or they should add this to their topics to research). Note that at the time of this writing, , vaccination requirements to attend public school are in flux, and students need to be aware of how regulations are changing and why.

Before closing the session, remind students that they should use the recommended textbooks for the course as a starting point for their research, and that Harrison’s Principles of Internal Medicine is also an excellent resource. Encourage them to use recent review articles as necessary to address their group learning objectives, but to limit their use of primary research articles to situations where they have an extremely specific question to answer.

Also remind students that they will be given specific review articles to read at the end of the second (B) session.

**STUDENT SESSION B**

It is the job of the group leader, not the facilitator, to decide when each step has been completed to the satisfaction of the group. The facilitator should help if asked, or if they feel that the group is not progressing toward completion in a timely manner. This session has 3 major segments.

*Review and modification of the group concept map (30-45 minutes)*

- The group must assemble a coherent version of a scientific concept map that incorporates all of the data that they compiled in session A, as well as any relevant information that they have gathered during their research.
- The leader (with help from the recorder and the facilitator, if necessary) should ensure that all of the group’s learning objectives have been resolved by the end of this segment of the session.
- The learning objectives created by the content experts should be revealed to the group at some point during this segment of the session in order to allow students to research and incorporate any elements of the content experts’ objectives that are missing from their concept map. We have used several methods to deliver the learning objectives, including having the learning objectives concealed from the facilitators and revealed electronically to the entire class at the same time. More commonly, the learning objectives are printed along with case and released to the group by the either the group leader or the facilitator at the point where the group believes that they have completed their concept map satisfactorily. Waiting until the group has addressed their own learning issues is important, as it keeps this session learner centered. As students’ concept mapping skills improve, they find that there is significant overlap between the content expert learning objectives and their own, and the stress over the timing of the release of the learning objectives decreases.

*SNAPPS^7^ presentation (by leader) (10-15 min)*

- The point of this presentation is to allow students to develop their clinical presentation skills in a low stake’s environment. The leader should identify when in the case the presentation is being made before starting the presentation. The most common choice is either before or after the lab results are obtained.
- After the 5-7 minute presentation, the group should provide constructive feedback. Once the peer feedback has been discussed, the facilitator can provide any additional feedback they feel is necessary.

*Presentation of case by the group (~60-80 minutes)*

- The group should present their concept map to each other, with the group and the facilitator providing feedback as necessary. This provides the group with the opportunity to modify the concept map and to rehearse presenting to the class. They should also develop a list of additional learning objectives if any concepts need clarification, and/or a list of questions that can be put to the content experts during the concept map review (“wrap up”) session.
- Everyone in the group must present a part of their concept map.
- The goal is for the class presentation to take no more than 25 minutes during the concept map review session. This B session rehearsal typically takes longer, as students adjust and improve their concept map as they present it.

**PBL Case of Unvaccinated Child with Measles Infection**

**REVIEW ARTICLES** (read before concept map review session)

Hübschen JM, Gouandjika-Vasilache I, Dina J. Measles. *The Lancet*. 2022;399(10325):678-690. doi:10.1016/S0140-6736(21)02004-3

Rota PA, Moss WJ, Takeda M, de Swart RL, Thompson KM, Goodson JL. Measles. *Nature Reviews Disease Primers*. 2016;2(1). doi:10.1038/nrdp.2016.49

#### **DULUTH PROBLEM BASED LEARNING (PBL) GOALS**

Students will:

1. Become proficient in developing a detailed concept map relating the basic and clinical science information regarding a specific disease.
2. Develop skills in relating clinical information to others in a concise and efficient manner.
3. Increase ability to work in teams with members who have different skills/knowledge base.

#### **MEASLES CASE LEARNING OBJECTIVES**

1. *Global epidemiology:* Contrast the characteristics of regional, national, and international measles outbreaks, including a comparison of the rates of long-term adverse events.
2. *Prodromal phase:* Explain the route of entry and sites for viral replication as they relate to the mechanisms of innate immunity.
3. *Eruptive and convalescent phases*: Differentiate among the pathophysiological processes that cause each of the clinical symptoms and signs experienced by a typical measles patient as the infection evolves over time.
4. *Adaptive immunity:*  Characterize the following elements of adaptive immunity: a) the relationship between MHC Class I expression and cell mediated toxicity; b) the separate roles for dendritic cells, lymphocytes, natural killer T cells and B cells in combating measles virus; c) the role of MHC class II expression in long-term humoral immunity; and d) the immunological functions of antibodies and the timing of the emergence of IgM, IgA, and IgG molecules during infection.
5. *Herd immunity:* Describe the impact of herd immunity on the estimation of infectivity (R value) and on the spread of measles in a population.
6. *Vaccine hesitancy:* Explain how the ethical principles of beneficence, non-maleficence, autonomy, and justice apply to vaccination decisions, and apply these four principles when communicating effectively with patients and their families about vaccines.
7. *Pharmacology*: Outline the mechanism of action for established measles treatments, focusing on acetaminophen’s antipyretic and analgesic actions, vitamin A’s effects on mucosal immunity and on the timing for administration of immune globulin.
8. **CONCEPT MAP REVIEW (“WRAP UP”) SESSION**

The purposes of this session are:

- To allow students to ask questions of the content experts, and to resolve any major differences among the groups’ concept maps
- To allow content experts to clarify any significant misconceptions with respect to the learning objectives
- To provide an opportunity for students to see how other groups handled the presentation and the concept mapping

The decision of the group to present should be made ahead of the session, so that the group can display/distribute their final map to the class before the session starts.

The facilitators should attend this session, although most of the faculty contributions will be made by the content experts.

This session has 3 major segments:

*SNAPPS^7^ presentation (5-10 minutes)*

- This serves as an introduction and allows the class to observe differences among groups with respect to how their leaders organized the clinical presentation. Constructive criticism should be provided to the presenter outside of the session time.

*Group concept map presentation (20-25 minutes)*

- The group presents their concept map to the class. Comments and questions should be limited during this time to allow time for general questions about the case and the learning objectives.

*Question period (25-35 minutes)*

- Students are invited to ask questions about the concept map, the learning objectives, and/or anything they need clarification on based on their research. The group members, content experts, or other faculty members can answer.

**REFERENCES FOR FACILITATORS**

1 Field RI, Caplan, AL. A Proposed Ethical Framework for Vaccine Mandates: Competing Values and the Case of HPV. Kenedy Institute of Ethics Journal. 2008;18; 111-124. <https://doi.org/10.1353/ken.0.0011>

2. Jacobson RM, Van Etta L, Bahta L. The C.A.S.E. Approach. Guidance for talking to vaccine-hesitant parents. *Minnesota Medicine*. 2013;April; 49-50.

3. Hübschen JM, Gouandjika-Vasilache I, Dina J. Measles. *The Lancet*. 2022;399(10325):678-690. doi:10.1016/S0140-6736(21)02004-3

4. Rota PA, Moss WJ, Takeda M, de Swart RL, Thompson KM, Goodson JL. Measles. *Nature Reviews Disease Primers*. 2016;2(1). doi:10.1038/nrdp.2016.49

5. Institute for Healthcare Improvement. Ask Me 3: Good Questions for Your Good Health. Accessed September 23, 2025. <https://www.ihi.org/library/tools/ask-me-3-good-questions-your-good-health>

6. Onello E, Friedrichsen S, Krafts K, Simmons G, Diebel K. First year allopathic medical student attitudes about vaccination and vaccine hesitancy. *Vaccine*. Published online November 2019. doi:10.1016/j.vaccine.2019.10.094

7. Nixon J, Wolpaw T, Schwartz A, Duffy B, Menk J, Bordage G. SNAPPS-Plus: An Educational Prescription for Students to Facilitate Formulating and Answering Clinical Questions. *Academic Medicine*. 2014;89(8):1174-1179. doi:10.1097/ACM.0000000000000362
