# Supplementary material for: Problem-Based Learning Case of Unvaccinated Child With Measles Infection: Integrating Viral Pathogenesis, Immunology, and Vaccine Ethics
Source: MedEdPORTAL. 2026 Feb 6;22:11577. doi: 10.15766/mep_2374-8265.11577 (PMC12890053; doi:10.15766/mep_2374-8265.11577)
Supplement: Supplementary file 1 — Faculty Guide.docxExam Questions.docxHow-to-Deliver Quick Guide.pdfHow-to-Deliver Full Guide.pdf [file mep_2374-8265.11577-s001.zip › B. Exam Questions.docx]

### **Problem Based Learning (PBL) Case of Unvaccinated Child with Measles Infection: Integrating Viral Pathogenesis, Immunology and Vaccine Ethics**

### **APPENDIX B: MULTIPLE CHOICE TEST QUESTIONS LISTED BY LEARNING OBJECTIVE**

Text in blue is intended as guidance for faculty members who are constructing a test.

Correct responses are preceded by a check mark, which should be removed before use. The correct response is also typed below the question, accompanied by a short rationale.

The average correct response frequency, expressed as a percentage, is followed by the number of correct responses over the total number of times a question was used in graded assessments. The average discrimination index^1^ for each question was calculated by the digital exam platform, ExamSoft^Ò^.

**REFERENCE****S**

1. Ebel RL, Frisbie DA. *Essentials of education measurement*, 4^th^ ed. Englewood Cliffs, NJ: Prentice Hall; 1986.

### **OBJECTIVE 1: GLOBAL EPIDEMIOLOGY**

#### Question 1 Average correct response frequency: 69% (91/131); Average discrimination index: 0.145

Despite the existence of an effective vaccination against measles virus, there is still significant mortality worldwide from measles infection. Of the estimates listed below, indicate which most closely represents the current mortality rate from measles globally

. A. 2 - 3 deaths per day

✓B. 200 - 300 deaths per day

C. 2,000 - 3,000 deaths per day

D. 20,000 - 30,000 deaths per day

Correct response: In 2023, 107,500 people (mainly children) died from measles, a rate of 296 deaths per day.

*We recommend updating this question annually, as global measles mortality rates change. However, the order of magnitude differences among the foils exceeds the variability in global measles fatality rates.*

#### Question 2 Average correct response frequency: 75% (97/129); Average discrimination index: 0.24

Which of the following statements is true about measles virus?

✓A. Before the development of a measles vaccine, measles infection was nearly universal in childhood

B. Measles is a paramyxovirus that is believed to have started infecting humans in the early 1800’s

C. Measles infection has an annual global mortality rate of <100 people in 2016

D. Nutritional status has little impact on the measles disease course or outcome

E. One of the most common complications of measles infection is vitamin C deficiency

Correct response: Before the development of a measles vaccine, measles infection was nearly universal in childhood.

References to measles infection can be found as early as the 7th century (i.e., measles is not a new human virus). Before vaccination, almost all people were infected with measles during childhood. Measles infections still occur in the developing world with a global fatality rate of roughly 80,000 – 100,000 deaths per year. Vitamin C deficiency is not a known ‘complication’ of measles (diarrhea, otitis media and pneumonia are). Nutritional status, if poor, can make a measles sufferer more vulnerable to secondary infection and to worse outcomes.

### **OBJECTIVE 2: PRODROMAL PHASE**

#### Question 3 Average correct response frequency: 70% (116/178); Average discrimination index: 0.055

The measles virus establishes an initial infection in epithelial cells of the upper respiratory tract. What is the most likely host-defense response to occur immediately following exposure to the virus but before widespread viremia?

A. Production of viral specific IgM in the circulation

B. Production of viral specific IgG in the circulation

C. Production of viral specific IgA in the mucosal lining of the upper respiratory tract

✓D. Expression of Type I interferons by infected epithelial cells of the upper respiratory tract

E. Expression of IL-4 by viral specific Th2 lymphocytes in lymph nodes

Correct Response: Expression of Type I interferons by infected epithelial cells of the upper respiratory tract. Option A is incorrect, as measurable IgM antibodies do not appear until the virus has begun replicating in the upper respiratory tract. Option B is incorrect, as a rise in measurable IgM antibodies must precede the appearance of IgG. Option C is incorrect, as a rise in measurable IgM antibodies must precede the appearance of IgA. Option D is True; the initial cellular response to viral penetration and replication is the production of Type I interferons. This step promotes MHC class I expression of viral antigen on the surface of viral infected cells to facilitate cell mediated clearance of measles virus proliferation. Option E is incorrect: IL-4 production is a late response corresponding to the latter stage of humoral defense and antibody isotype switching.

#### Question 4 Average correct response frequency: 85% (170/220); Average discrimination index: 0.175

A patient has been referred to your clinic by the school nurse for suspicion of measles. This 13-year-old male reports feeling ill, with fever, cough, and conjunctivitis. He did not receive the MMR vaccine, and he has just returned to school today following a family trip to India. You check his mouth and see Koplik spots on the mucosal surface. You diagnose measles and then you inform the appropriate public health authorities. Based on these data, when was this patient most likely infected with the measles virus?

A. 10 - 13 hours ago

B. 1 - 3 days ago

✓C. 10 - 13 days ago

D. 21 –23 days ago

E. >30 days ago

Correct response: 10 - 13 days ago. This test question pertains to the measles PBL learning objectives. In the PBL on measles infection in an unvaccinated patient, students develop a timeline of infection. Signs and symptoms of cough/conjunctivitis/fever and Koplik spots occur 10 - 13 days following initial infection.

#### Question 5: Average correct response frequency: 81% (106/131); average discrimination index: 0.205

A 5-year-old boy is admitted to the emergency room accompanied by his mother. The mother has a concern about a rash that has been spreading from the boy's face to the trunk over the last day. The appearance of the rash was preceded by fever, cough, coryza, and conjunctivitis. Up to now the child's health has been unremarkable, though the patient has not received any vaccinations. The physical examination is significant for a mild fever, Koplik spots in the oral cavity, and a morbilliform rash on the face and trunk.

Based on the patient's history and presentation, in which Baltimore Class (see figure below) is the infectious agent most likely responsible for the child's symptoms?


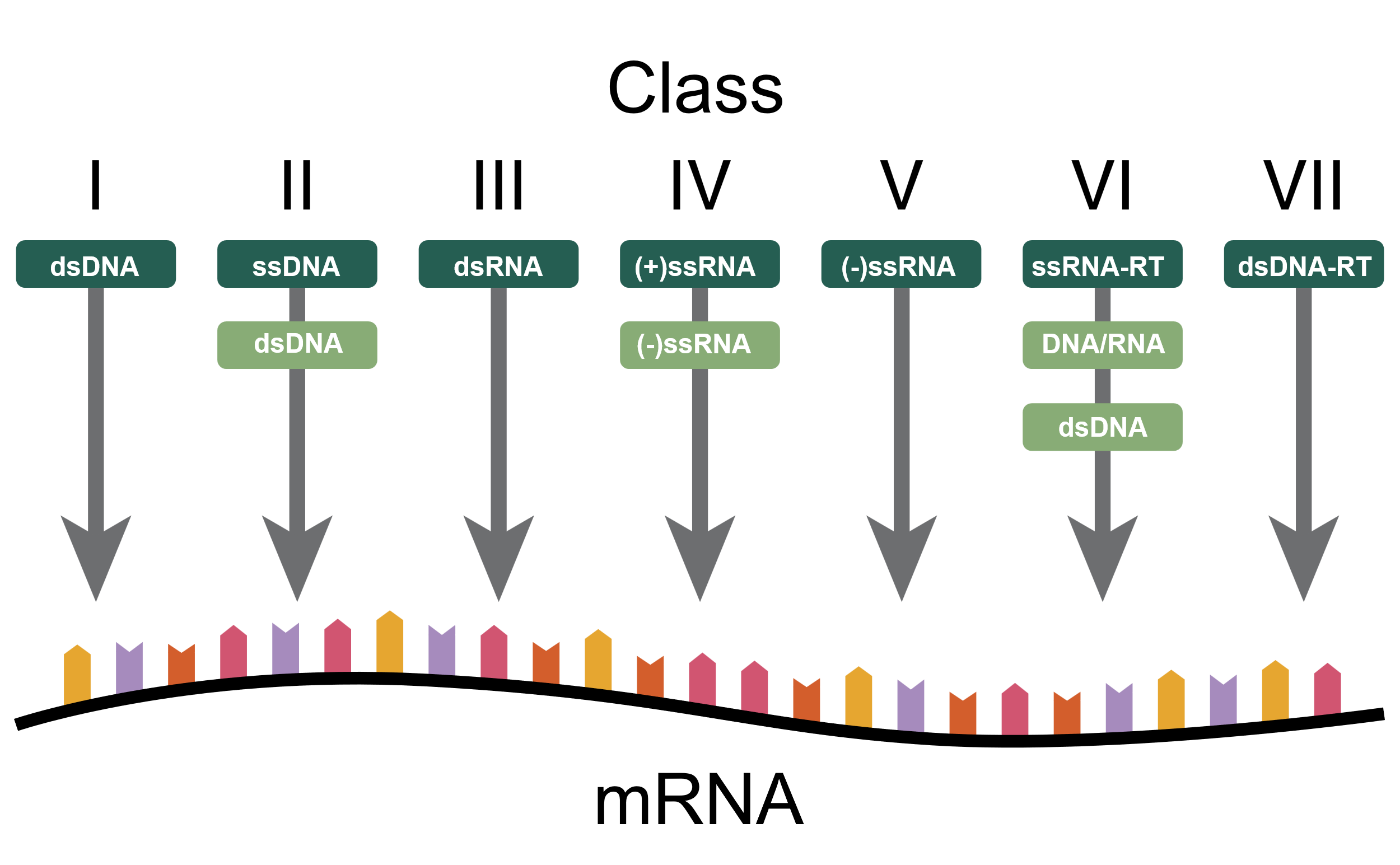


Figure B1: Baltimore classification of viruses.
Image by Splettstoesser, T, retrieved from <https://commons.wikimedia.org/wiki/Category:Baltimore_classification#/media/File:VirusBaltimoreClassification.svg> on September 23, 2025. Image is in the public domain.

A. Group I: DNA (+/-)

B. Group II: DNA (+)

C. Group III: RNA (+/-)

D. Group IV: RNA (+)

✓E. Group V: RNA (-)

F. Group VI: RNA (+)

G. Group VII: DNA (+/-)

Correct response: Group V: RNA (-). The clinical presentation in an unvaccinated child is consistent with measles. Measles is caused by a negative strand RNA virus that follows the gene expression strategy of the Baltimore Group V.

#### Question 6: Average correct response frequency: 85% (154/181); average discrimination index: 0.213

The incubation period for measles is 8 days consisting of viral replication and onset of viremia. This is followed by the prodrome period (days 8-14) characterized by syncytia formation, epithelial necrosis, fever, cough, coryza, conjunctivitis and appearance of Koplik spots; the appearance of maculopapular rash (days 14-21); and ultimately recovery (days 22 and beyond). What is the most likely host-defense response to occur in unvaccinated individuals immediately following exposure to this virus?

A. Production of viral specific IgM in the circulation

B. Production of viral specific IgG in the circulation

C. Production of viral specific IgA in the mucosal lining of the upper respiratory tract

✓D. Expression of Type I interferons by infected epithelial cells of the upper respiratory tract

E. Expression of IL-4 by viral specific Th2 lymphocytes

Correct response: Expression of Type 1 interferons by infected epithelial cells of the upper respiratory tract. Option A is incorrect; measurable IgM antibodies do not appear until virus has begun replicating in the upper respiratory track. Option B is incorrect; a rise in measurable IgM antibodies must precede the appearance of IgG. Option C is incorrect; a rise in measurable IgM antibodies must precede the appearance of IgA. Option D is true; the initial cellular response to viral penetration and replication is the production of Type I interferons. This step promotes MHC class I expression of viral antigen on the surface of viral infected cells to facilitate cell mediated clearance of measles virus proliferation. Option E is incorrect; IL-4 production is a late response corresponding to the latter stage of humoral defense and antibody isotype switching.

### **OBJECTIVE 3: ERUPTIVE AND COVALESCENT PHASES**

#### Question 7 Average correct response frequency: 64% (119/186); average discrimination index: 0.26

In measles infection, what is the typical order of symptom appearance?

✓A. Fever, Koplik spots on oral mucosa, rash

B. Fever, rash, Koplik spots on oral mucosa

C. Koplik spots on oral mucosa, fever, rash

D. Koplik spots, rash, fever

E. Rash, fever, Koplik spots on oral mucosa

Correct response: Fever, Koplik spots on oral mucosa, rash

#### Question 8: Average correct response frequency: 65% (42/65); average discrimination index: 0.32

A patient has been referred to your clinic by the school nurse for suspicion of measles. This 13-year-old male reports feeling ill, with fever, cough, and conjunctivitis. He did not receive the MMR vaccine and has returned to school today following a family trip to India. You check his mouth and see Koplik spots on the mucosal surface; a rash is not present. You diagnose measles and then you inform the appropriate public health authorities.

Based on this clinical presentation, when would you expect serologic testing for measles IgG to **begin** to reveal *elevated levels* of IgG?

A. 4 - 7 days prior to this presentation

B. now

✓C. 5 - 15 days from now

D. 45 - 60 days from now

E. >90 days from now

F. Measles IgG will not appear because measles virus has suppressed the immune response

Correct response: 5 to 15 days from now. The patient's presentation (fever, cough, conjunctivitis, Koplik spots but absence of skin rash) is consistent with an infection occurring 10-13 days ago. In the PBL on measles infection in an unvaccinated patient, students develop a timeline of infection and the immune responses including immunoglobulin production. A rise in IgG is typically evident starting around Day 18 after initial measles infection. For this patient, the IgG detection should occur approximately 5 -15 days from his presentation to the school nurse. Though measles does suppress the immune response during active infection, the host can still make immunoglobulin IgM and IgG.

### **Objective 4: Adaptive Immunity**

#### Question 9 Average correct response frequency: 90% (190/220); average discrimination index: 0.100

Children with agammaglobulinemia recover from measles infection and develop lifelong immunity. Children with defects in T cell function have great difficulty recovering from a measles infection. Which immune response is the most important for patient survival when infected with measles?

A. Elevated levels of virus specific IgA levels block virus invasion

B. Increased neutrophil-mediated inflammation promotes flushing of viruses from tissues

✓C. MHC class I directed cytotoxic T-cell mediated apoptosis

D. Antibody neutralization and clearance of viral particles

E. Antibody dependent cell mediated cytotoxicity

Correct Response: MHC class I directed cytotoxic T-cell mediated apoptosis. Option A is incorrect; Virus specific IgA will be absent. Option B is incorrect; Neutrophils do not contribute a defense activity to measles infection. Option C is true; cell mediated Immunity is still functional in the absence of virus specific antibody. Option D is incorrect; no Measles specific antibody is present. Option E is incorrect; no Measles specific antibody is present

#### Question 10 Average correct response frequency: 93% (185/199); average discrimination index: 0.15

Often, people perceive that protection against viruses, such as measles, relies solely on the production of immunoglobulins, such as IgG. However, children with agammaglobulinemia recover from measles infection while children with defects in T cell function have great difficulty recovering from infection. Which immune response is most important for patient survival?

A. Elevated levels of virus-specific IgA levels block virus invasion

B. Increase Neutrophil-mediated inflammation promotes flushing of viruses from tissues

✓C. MHC class I directed Cytotoxic T-cell mediated apoptosis

D. Antibody neutralization and clearance of viral particles

E. Antibody-Dependent Cell-Mediated Cytotoxicity

Correct response: MHC class I directed Cytotoxic T-cell mediated apoptosis

#### Question 11 Average correct response frequency: 93% (113/122); average discrimination index: 0.04

Vaccination with attenuated measles virus produces long term protection that exceeds 10 years by stimulating which of the following responses?

A. Expressing virus antigen in MHC class I complexes to bind cytotoxic T cells

B. Producing large populations of B cells producing antibody specific to the measles virus

C. Producing large populations of cytotoxic T cells specific to measles antigen displayed by MHC class I molecules

D. Producing large populations of plasma cells that produce high affinity antibodies to the measles virus

✓E. Production of a population of memory B cells that produce antibody to the measles virus

Correct Response: Production of a population of memory B cells that produce antibody to the Measles virus

### **OBJECTIVE 5: HERD IMMUNITY**

#### Question 12 Average correct response frequency: 98% (108/115); average discrimination index: 0.000

Which of the following best describes the principle of herd immunity?

A. Early life exposure to zoonotic infections, such as bovine viruses, confers protection in later life to human population

✓B. Once a critical mass of people acquires protective immunity, either through vaccination or infection, they can serve as a buffer for the rest

C. Only large-scale vaccination campaigns are effective at eradicating infectious disease

D. Once the majority of the adult population has been vaccinated against a communicable disease, their offspring do not need vaccination

E. A concept used by vaccine opponents to argue that vaccines are unnecessary and dangerous

Correct response: Once a critical mass of people acquires protective immunity, either through vaccination or infection, they can serve as a buffer for the rest. Herd Immunity is described as the decreased chance that an individual who is susceptible to an infection will become sick due to the surrounding number of individuals in the population who are immune to the infection (and therefore will not carry or spread the infection). The other responses do not describe herd immunity concept.

### **OBJECTIVE 6: ETHICAL PRINCIPLES AND VACCINE HESITANCY**

#### Question 13 Average correct response frequency: 89% (58/65); average discrimination index: 0.11

You are a family physician. You have decided that you no longer will care for families who refuse to vaccinate. The reason for your decision is that you do not want to put your other vulnerable patients (immune-suppressed, unvaccinated) in harm’s way should a vaccine-preventable disease be brought into your clinic from a vaccine-hesitant family.

In making this decision on behalf of your larger patient population, you are invoking the following ethical principle most prominently:

A. Autonomy

B. Beneficence

C. Justice

✓D. Non-maleficence

Correct response: non-maleficence. The optimal choice is non-maleficence because the physician is basing the decision on *reducing harm or risk of harm* to the other patients in the physician’s practice. Reducing harm is non-maleficence. Autonomy is generally used to refer to the patient’s right to make decisions about their own body, not to a presumed right of the physician to run their practice as they choose.

#### Question 14 Average correct response frequency: 57% (89/157); average discrimination index: 0.29

You are a family physician. You have a pediatric patient whose parents are adamant that he not be vaccinated. The unvaccinated child represents a potential risk to other patients in your clinic who may be immunocompromised. Despite this risk, you choose to keep seeing the patient because other health care options are limited in the community. During future visits, you decide to apply motivational interviewing techniques to encourage the parents to vaccinate the patient.

In making this decision on behalf of your pediatric patient, you are invoking the following ethical principle most prominently:

A. Autonomy

✓B. Beneficence

C. Justice

D. Non-maleficence

Correct response: Beneficence. The physician in this case is representing the best interest of the child and demonstrates the principle of beneficence.

### **OBJECTIVE 7: PHARMACOLOGY AND TREATMENT**

#### Question 15 Average correct response frequency: 66% (85/129); average discrimination index: 0.27

You are working in your rural hospital’s Emergency Room on a Thursday evening. Your patient is a 5-month-old infant girl who arrives with her mother. The patient is breastfeeding robustly; no fevers, irritability or other symptoms are reported. However, the mother anxiously reports that her infant daughter was exposed to measles virus at a family event on Sunday, 4 days ago. You are aware of a measles outbreak reported in your region. The physical examination is unremarkable, and the child appears to be in good health. Which of the following courses of action is the most appropriate?

A. recommend MMR (measles-mumps-rubella) vaccination via intramuscular route

B. recommend blood test to check for HIV infection

C. obtain blood, urine, and cerebrospinal fluid for measles culture and PCR testing

✓D. recommend the administration of immunoglobulin (Ig)

E. request that your patient’s mother call your state Department of Health to report an impending case of measles

Correct response: recommend the administration of immunoglobulin (Ig), This is a challenging question, as minimal information is provided and the test-taker must make some clinical judgments. However, the clinical dilemma centers around a probable case of measles exposure in an unvaccinated infant (MMR vaccine not given until >12 months of age). General guidelines suggest that if exposure to measles is less than 72 hours (3 days), then MMR vaccination is recommended. However, if exposure to measles is more than 72 hours, then injection of intramuscular Ig (immunoglobulin) is recommended.

Option A is incorrect, as MMR is not recommended > 72 hours post-exposure (this case exposure is 96 hours prior). Additionally, MMR is given via a subcutaneous route, not intramuscular. Option B is incorrect, as HIV testing is not necessary since there is no history to suggest immune system impairment. Option C is incorrect since extensive testing for measles is not needed in an asymptomatic patient. As noted, Option D is correct since the infant was exposed > 72 hours ago and passive immunity from immunoglobulin is needed to reduce risk of measles infection. Option E is incorrect because the obligation to report any infectious disease rests with the healthcare provider/physician. Also, active disease is what is reported.

#### Question 16 Average correct response frequency: 68% (87/128); average discrimination index: 0.215

A patient has never had the measles virus or the measles vaccination. This patient is inadvertently exposed to measles. Following this exposure, what is the time frame during which the Measles-Mumps-Rubella (MMR) vaccine can protect this patient from getting measles infection?

A. Up to 24 hours

B. Up to 48 hours

✓C. Up to 72 hours

D. Up to 96 hours

E. The MMR vaccine will not protect this patient from getting measles infection.

Correct response: Up to 72 hours. Current guidelines suggest that the MMR vaccine can protect if given within 72 hours of measles exposure. After 72 hours, the patient would need passive immunity with IgG.
