# Supplementary material for: Problem-Based Learning Case of Unvaccinated Child With Measles Infection: Integrating Viral Pathogenesis, Immunology, and Vaccine Ethics
Source: MedEdPORTAL. 2026 Feb 6;22:11577. doi: 10.15766/mep_2374-8265.11577 (PMC12890053; doi:10.15766/mep_2374-8265.11577)
Supplement: Supplementary file 1 — Faculty Guide.docxExam Questions.docxHow-to-Deliver Quick Guide.pdfHow-to-Deliver Full Guide.pdf [file mep_2374-8265.11577-s001.zip › C. How-to-Deliver Quick Guide.pdf]

MEASLES PBL: HOW TO DELIVER

Quick Guide, Appendix C

1 Determine logistics (curriculum time, dates, room locations, faculty facilitators and content experts)

- Recruit faculty and facilitators (Ideally one facilitator per 6-8 person small group)
- Reserve on-campus/virtual meeting rooms

2 PART 1: Faculty preview of the case

1 hour

- Content experts and course directors review and update the case
- → Faculty facilitators attend a one-hour preview session to review the updated case

3 PART 2: Student Session A

2 hours

- Each facilitator guides 6-8 students in a small group
- → Students receive details about a clinical case incrementally while concurrently developing a CONCEPT MAP
- → Students collectively identify their knowledge gaps and assign homework to each student to research these knowledge topics
- → Students prepare a presentation about what they learned from the homework exercise and present this to the group at Session B (below)

4 PART 3: Student Session B

2 hours

- A designated student presents a short (3-5 mins) clinical case summary
- → Students integrate the "homework" knowledge gained and collectively enhance and discuss the CONCEPT MAP

5 PART 4: Wrap Up Session

1 hour

- Randomly selected small group presents their concept map to the whole class
- → The whole class discusses the concept map and modifies

6 Student Assessment

Content experts used MCQs and subsequent item analysis to assess student competency with respect to the case learning objectives

See Appendix D for an in depth "How to Deliver" Guide

Medical School, Duluth Campus
University of Minnesota
Driven to Discover
