# Supplementary material for: Problem-Based Learning Case of Unvaccinated Child With Measles Infection: Integrating Viral Pathogenesis, Immunology, and Vaccine Ethics
Source: MedEdPORTAL. 2026 Feb 6;22:11577. doi: 10.15766/mep_2374-8265.11577 (PMC12890053; doi:10.15766/mep_2374-8265.11577)
Supplement: Supplementary file 1 — Faculty Guide.docxExam Questions.docxHow-to-Deliver Quick Guide.pdfHow-to-Deliver Full Guide.pdf [file mep_2374-8265.11577-s001.zip › D. How-to-Deliver Full Guide.pdf]

MEASLES PBL: HOW TO DELIVER

Full Detailed Guide, Appendix D

This document (Appendix D) is a guide for the delivery of this PBL case (Appendix A).

BACKGROUND

This case scenario (Appendix A) describes a partially immunized toddler who presents to a rural family medicine clinic after international travel. The case follows the patient and an unvaccinated cousin, culminating with the patient returning for a pre-kindergarten physical, while still missing several required immunizations. Clinical and basic science faculty content experts developed the learning objectives, collaborated to write and review multiple choice questions (MCQs; Appendix B) for each learning objective, and offered formative feedback to the students regarding their final concept maps. Session facilitators were primarily basic scientists who had 6-32 hours of training and/or experience in the PBL approach, but not specific expertise in viral diseases or immunology.[1]

At the point in the curriculum where this case was delivered, students had already developed skills in the PBL process by participating in 5-10 other cases. All of our campus's PBL cases shared the common goals of improving student proficiency in the following:

Developing clinical concept maps	Presenting clinical information	Working in teams

Students received formative feedback on these common objectives from their facilitators during this and all other PBL cases in the curriculum.

IMPLEMENTATION

Materials:

- Electronic educational delivery platform format (e.g., Canvas©) or paper version of the case
- Meeting space for groups of 6-8 students
- Colored marker set for each small group
- Large group classroom space for a review session with whiteboards
- Only two students attend the first student session with a personal laptop, tablet, or other device
- Every student may attend the second student session with a personal laptop, tablet, or other device

Personnel:

- Small group facilitators, 1 per group
- Faculty Content Experts, to review each case learning objective, assess student competencies and provide formative feedback with respect to specific educational objectives

1 **Determine logistics** weeks to months in advance (curriculum time, dates, room locations, faculty facilitators and content experts)

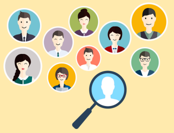

Recruit faculty and facilitators (Ideally one facilitator per 6-8 person small group)

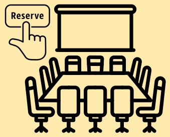

Reserve on-campus/virtual meeting rooms

2 **PART 1:** Faculty preview of the case

1 hour

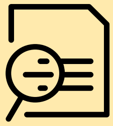

Content experts and course directors review and update the case

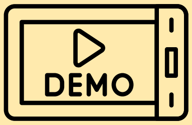

Faculty facilitators attend a one-hour preview session to review the updated case. During the preview session, the facilitators are reminded of the case learning objectives and encouraged to ensure that student discussions include explorations of all 7 major objectives, represented in Figure 1.

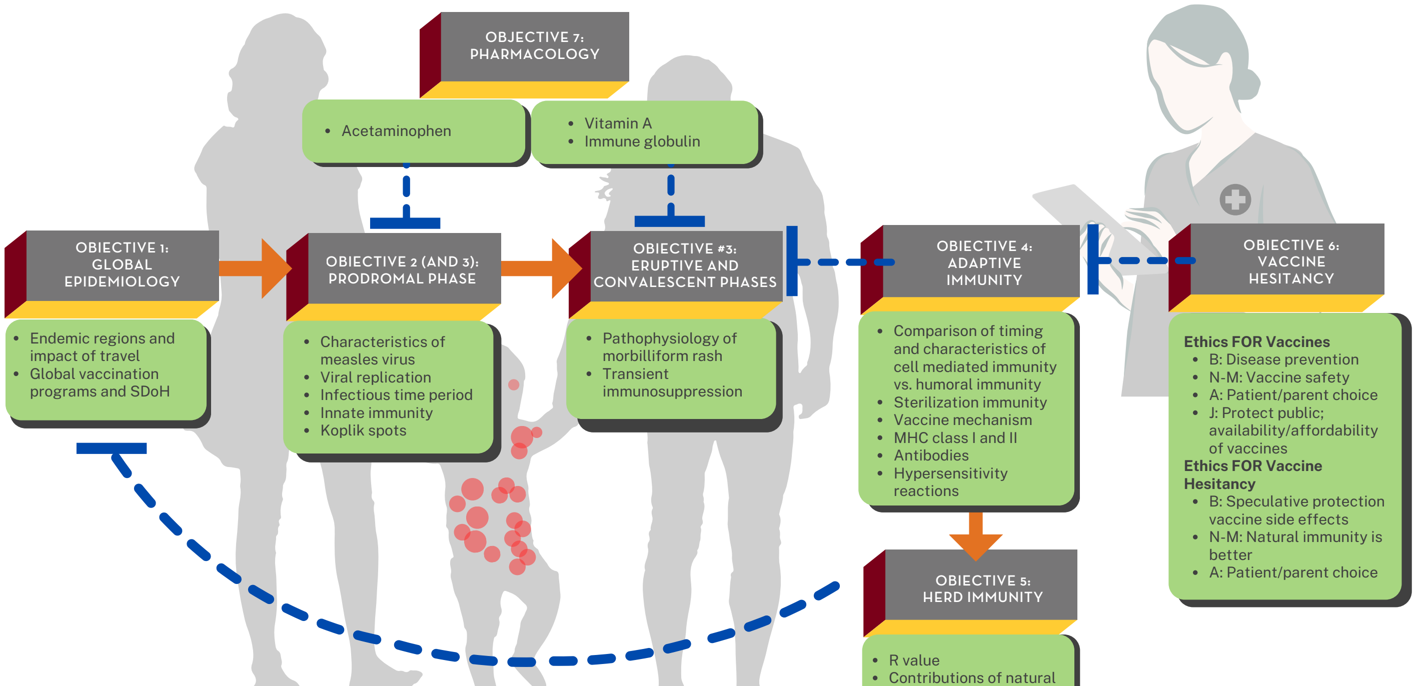

Figure 1.

Figure 1. Content expert overview of measles concept map organization. Student scientific concept maps should be organized into 7 major interlinked sections, each corresponding to a specific learning objective for the case. The distribution of key topics (indicated as bullet points) is not evenly divided among the objectives as adaptive immunity (objective 4) is the core focus of the case and should integrate across several sections of the map. Actual student scientific concept maps should contain much greater detail on each key topic. Orange arrows indicate causation; blue blunt ended dashed lines indicate inhibition. SDoH=social determinants of health; MHC=major histocompatibility complex; R value=R-naught or R0 value for communicable diseases; B=beneficence; N-M=non-maleficence; A=autonomy; J=justice.

3 PART 2: Student Session A

2 hours

Each facilitator guides 6-8 students in a small group. Only 1-2 students may use a computer or device during this session (to take group notes and/or look up definitions)

Students receive details about the clinical case incrementally while documenting new data as they receive it. Concurrently, they develop their scientific CONCEPT MAP

Students identify gaps in their scientific concept map that need to be researched before the next session

Students will prepare a presentation about what they learned from the homework exercise and present this to the group at Session B (below)

Students read through the following 9 sections of the case, stopping to respond to the prompts included in each section.

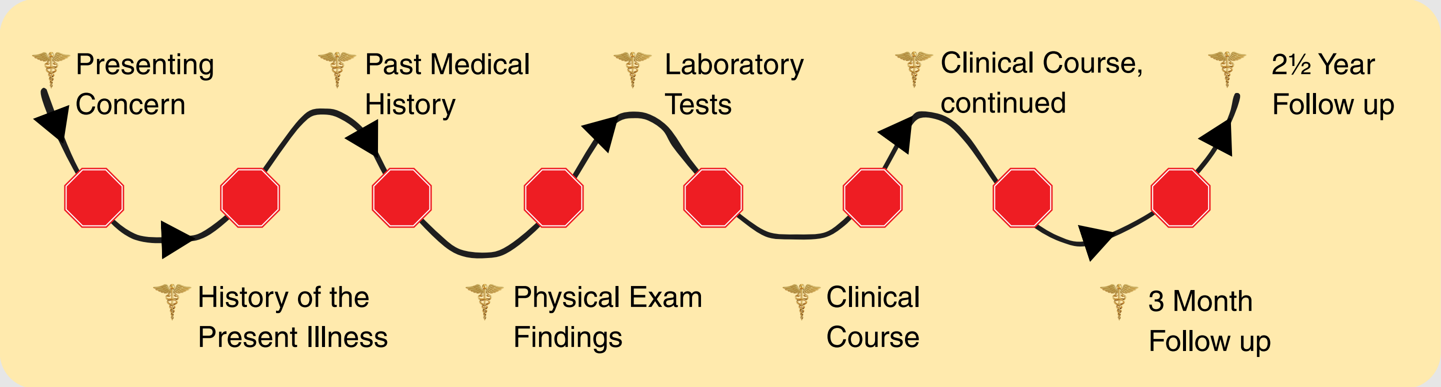

= Stop and discuss

- Students should not move on to the next section until they have answered the question prompts in the Student Guide and added information to their group's CONCEPT MAP.
- At the conclusion of Session A, students collectively identify their knowledge gaps and assign homework topic(s) to each member.

Two examples of the Session A discussion process are shown below, representing a small part of the larger PBL discussion process during Session A.

At the end of the Presenting Concern section, the students should have a data list and draft concept map that are similar to those represented below. The draft map example indicates that the students' initial draft concept map could include infection, allergic reactions and teething as the potential cause of at least one of the patient's symptoms.

DATA LIST	
**Normal**	**Abnormal**
2 yo	Fever
Male	Rash

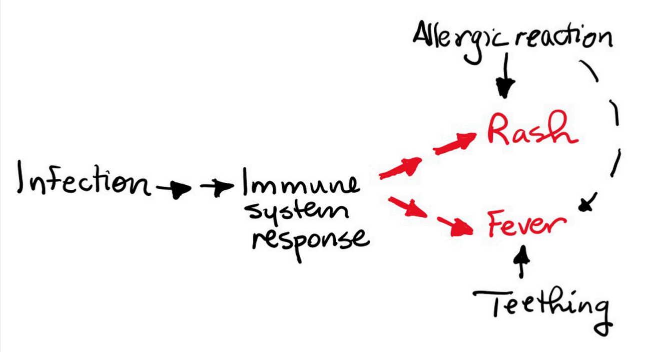

At the end of the History of Presenting Illness section, the students' data list should be similar to the table shown below. Data bolded in red appear in the subsequent concept map example.

DATA LIST		
**Normal**	**Abnormal**	**Lab results, treatments and procedures**
2 yo	**Fever (<103°F axillary); worse in evenings**	**Acetaminophen**
Male	Spreading rash from cheeks to body	
Rural MN	Clingy behaviour	
Recent travel to India and Malaysia 1 week ago Drank bottled water	Avoiding solid foods Decreased appetite, Diarrhea in Malaysia	
Normal breast feeding	**Irritability**	
Normal urine and tear production	Watery eyes	
No rigors or lethargy	Dry cough	
No mouth sores	Non-bloody emesis (2x)	
Clinic waiting room	Diarrhea (2x)	

By the end of session A, the student draft concept map should include an exploration of the prodromal phase and early measles infection (example to the right). The green circled items represent separate medical school courses where relevant content had been previously taught (e.g., “Neuro” & “Foundations”). The students' concept map should incorporate their accumulated knowledge.

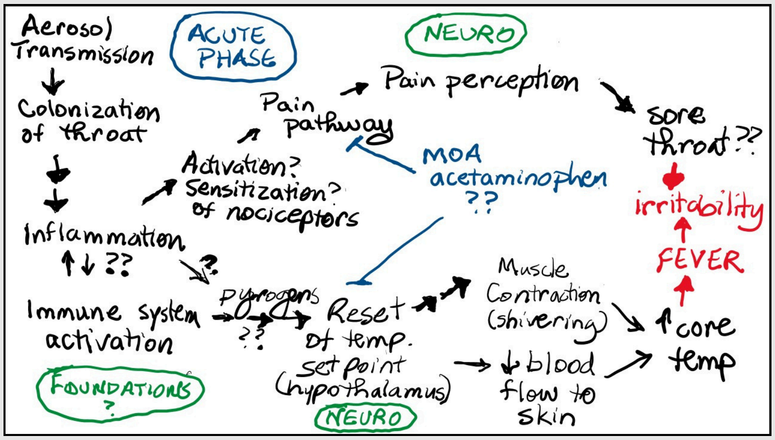

4 PART 3: Student Session B

2 hours

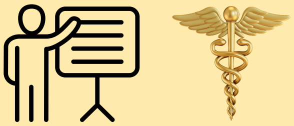

A designated student presents a short (3-5 mins) clinical case summary

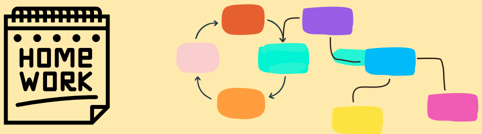

Students integrate their "homework" into the collective CONCEPT MAP and present the completed map to each other. The facilitator ensures that faculty learning objectives are successfully incorporated into the final group map

At our institution, we used the SNAPPS[2] format to: Summarize, Narrow, Analyze, Probe, Plan & Select. In this method or equivalent, the students practice presenting and listening to a clinical case while identifying the salient care issues.

The facilitators provide students with the faculty core learning objectives (Appendix A) and two topical review articles.[3,4]

While assessment of concept maps is beyond the scope of this guide, facilitators should look for the following in terms of a high-quality scientific concept map:

1. Breadth and Depth: A scientific concept map should explain the relationships among all of the data provided in the case (e.g., symptoms, lab results, public health requirements and laws, etc.), at the depth expected of a medical student.

2. Embeddedness & Interconnectedness: Using arrows or lines, the map should demonstrate how the aspects of the case interrelate and connect (for example, how the upper respiratory symptoms relate to viral invasion of the host mucosal airway tissues). The concept map should appear as one coherent figure. Information should not appear in multiple places on the map, and there should not be any lists.

3. Aesthetic presentation: The map should not appear as a ‘tangled mess’ to the observer, but rather an organized and integrated figure. Font size should not be too small to read easily. Text should be succinct and not too wordy.

4. Creative work: The map should not be composed of large chunks of ‘cut and pasted’ materials from other sources. Students should summarize and simplify external materials to fit efficiently into the concept map they are creating together.

5 **PART 4:** Wrap Up Session

1 hour

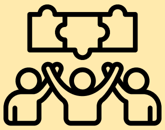

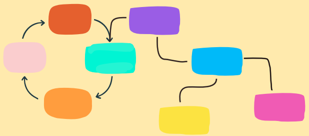

A randomly selected small group presents their CONCEPT MAP to the entire class, as well as to facilitators and content experts. The presentation should take no more than 1/2 of the allotted time

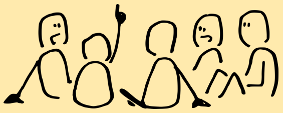

Everyone discusses the group map and any discrepancies between the groups are resolved

Students and faculty provide formative feedback to the selected group about their scientific map, noting strengths and weaknesses in the design and concepts.

Faculty content specialists are on hand to clarify, teach and ensure that students come away from the session with a correct understanding of the topics related to the case learning objectives.

Facilitators provided formative feedback to the group members at all stages of the concept mapping process, generally commenting on the appropriateness of student learning issues, and encouraging students to create a robust concept map with logical connections between pathological processes, symptoms and therapeutic interventions.

6 **Student Assessment**

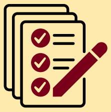

Content experts used MCQs and subsequent item analysis to assess student competency with respect to the case learning objectives

References

1. Zeitz HJ, Paul H. Facilitator expertise and problem-based learning in PBL and traditional curricula. Academic Medicine. 1993;68(3):203-204. doi:10.1097/00001888-199303000-00008

2. Nixon J, Wolpaw T, Schwartz A, Duffy B, Menk J, Bordage G. SNAPPS-Plus: An Educational Prescription for Students to Facilitate Formulating and Answering Clinical Questions. Academic Medicine. 2014;89(8):1174-1179. doi:10.1097/ACM.0000000000000362

3. Hübschen JM, Gouandjika-Vasilache I, Dina J. Measles. The Lancet. 2022;399(10325):678-690. doi:10.1016/S0140-6736(21)02004-3

4. Rota PA, Moss WJ, Takeda M, de Swart RL, Thompson KM, Goodson JL. Measles. Nature Reviews Disease Primers. 2016;2(1). doi:10.1038/nrdp.2016.49
